# Bioinformatics Analysis of microRNAs Associated with Metastatic Potential in Breast Cancer

**DOI:** 10.3390/biology15080617

**Published:** 2026-04-14

**Authors:** Aleksandra Nikezić, Sanja Goč, Jovana Stevanović, Miloš Brkušanin, Olgica Nedić, Jovana Jovankić, Zorana Dobrijević

**Affiliations:** 1 Department of Biology and Ecology, Faculty of Science, University of Kragujevac, 34000 Kragujevac, Serbia; aleksandra.nikezic@pmf.kg.ac.rs (A.N.); jovana.jovankic@pmf.kg.ac.rs (J.J.); 2 Institute for the Application of Nuclear Energy, University of Belgrade, 11080 Belgrade, Serbia; sanjagoc@inep.co.rs (S.G.); jovana.stevanovic@inep.co.rs (J.S.); olgica@inep.co.rs (O.N.); 3 Centre for Human Molecular Genetics, Faculty of Biology, University of Belgrade, 11158 Belgrade, Serbia; milosb@bio.bg.ac.rs

**Keywords:** microRNA, breast cancer, invasion, epithelial–mesenchymal transition, migration, metastasis, miR-222-3p, miR-205-5p, miR-141-3p, miR-200c-3p

## Abstract

Dysregulation of microRNA expression in breast cancer (BC) has been associated with molecular disturbances involved in cancer initiation, progression and metastasis. Specific microRNAs also act as endocrine modulators in BC, thereby influencing the biological behavior of the tumor and drug responses. Our objective was to employ bioinformatics tools to identify and characterize microRNAs acting as candidate players involved in epithelial–mesenchymal transition, migration, invasion, and/or hormonal regulation in BC. We systematically integrated microRNA profiling data from three different studies on BC cell lines with different invasive capabilities and from another study on lymph node metastases and matching primary BC, resulting in five microRNA hits—DE-microRNAs miR-146a-5p, miR-222-3p, miR-205-5p, miR-141-3p and miR-200c-3p. This set of microRNAs was evaluated for clinical significance in BC and subjected to target prediction, microRNA–mRNA network construction, functional enrichment analysis and quantification in BC cell lines by qPCR. An upregulated DE-microRNA, miR-222-3p, displayed distinctive pro-metastatic features, supported by its clinical relevance, as well as by the results of the functional enrichment analysis of its target genes. Downregulation of the members of the miR-200 family and miR-205-5p were significantly associated with negative clinical features, while their targets were enriched with genes that were relevant to cancer aggressiveness. These results are in line with the presumed functional relevance of the selected DE-microRNAs in BC.

## Simple Summary

Key microRNA players that are relevant for breast cancer aggressiveness, with crucial roles in mediating cancer migration, epithelial–mesenchymal transition, invasion and metastasis, remain underexplored. Therefore, we aimed to address this issue and uncover the potential candidate microRNAs associated with the metastatic potential of breast cancer by employing an in silico approach. We combined data from microRNA profiling studies to identify a minimal set of dysregulated microRNAs and to bioinformatically characterize their potential functional properties. Five microRNAs emerged as plausible cancer aggressiveness-associated regulators, one of which displayed distinctive pro-metastatic properties, while three downregulated microRNAs were significantly associated with negative clinical features, and their targets were enriched with genes that were relevant for cancer’s aggressiveness. These results are consistent with the presumed functional relevance of selected DE-microRNAs in BC and hold their value for future investigations focusing on biomarker discovery, improvements in diagnosis, outcome prediction, decision-making and treatment monitoring, as well as for enhancing therapeutic effectiveness.

## 1. Introduction

Breast cancer (BC) is the most common malignancy in females worldwide, with increasing incidence, and the number of new cases are predicted to rise by more than 35% by 2050, compared to the latest reports referring to 2022 [1,2]. According to the GLOBOCAN report for 2022, BC also accounts for around 17% of cancer-related deaths in women, exceeding other common highly lethal malignancies, including lung, colorectal and cervical cancer [1]. Triple-negative breast cancer (TNBC), which accounts for about 10–20% of BC cases and lacks receptors for estrogen, progesterone, and HER2, typically shows aggressive clinical behavior, arises at an earlier age, and is associated with a poorer prognosis [3,4].

Alarming statistics have positioned BC as a leading female health issue on a global scale, which has led to focusing efforts and resources towards BC prevention, early diagnosis, and identification of major molecular players involved in disease initiation and progression, which is relevant for designing advanced therapeutic approaches. However, despite progress in deciphering molecular pathways and key regulators involved in processes underlying the aggressive BC phenotype, many aspects of BC pathogenesis, metastasis, and recurrence remain unknown. Therefore, disentangling the complex network of molecular interactions that are relevant to BC aggressiveness holds promise for therapeutic target identification, as well as for molecular phenotyping and personalization of BC management, cancer therapy monitoring and detection of recurrent BC [5,6].

A growing body of evidence suggests that certain microRNAs exhibit oncogenic and/or tumor-suppressive properties, which has attracted the attention of researchers in the field of molecular carcinogenesis to this versatile class of regulatory RNAs [7]. By acting mostly through posttranscriptional regulatory mechanisms, these small (~21 nt long), single stranded RNAs incorporated into the RNA-induced silencing complex (RISC), modulate the expression of major oncogenes and tumor suppressors that are relevant to BC pathogenesis. Therefore, changes in microRNA profiles contribute to the dysregulation of gene expression associated with BC hallmarks, including cell proliferation, apoptosis evasion and invasion [8]. However, key microRNA players that are relevant to BC aggressiveness, with crucial roles in mediating cancer migration, epithelial–mesenchymal transition (EMT), invasion, and metastasis, remain underexplored. The discovery of such microRNA genes, their major targets and cancer-related mechanisms could aid in the identification of valuable novel BC biomarkers, as well as in designing advanced prognostic/surveillance algorithms and targeted therapeutic approaches.

In an attempt to uncover potential candidate microRNAs associated with BC metastatic potential and aggressive phenotypes, we used an in silico approach which combined data from microRNA profiling studies in primary vs. metastatic BC, and cell lines that are relevant to BC invasiveness. Specifically, we collected microRNA profiling results from the low-invasive BC cell line MCF7 and TNBC cell lines MDA-MB-231 and MDA-MB-468, as well as derivatives of the control cell line with a silenced estrogen receptor (ER) and increased invasiveness. We aimed to identify a minimal set of microRNAs from the intersection of dysregulated sets from multiple eligible studies, assess their potential clinical relevance in BC and analyze the correlation with the expression of mRNAs, based on the data from The Cancer Genome Atlas (TCGA). By identifying their potential target genes, we prepared data entries for the bioinformatics characterization of the potential functional properties of these microRNAs through gene ontology (GO), pathway, and cancer hallmark enrichment analysis. Furthermore, we sought to examine a microRNA–mRNA interaction network organized around our resulting BC signature microRNA set and to integrate the mRNA profiling data, which would enable the identification of hub genes and dysregulated microRNA targets that are relevant to BC EMT, invasion, and metastasis.

## 2. Materials and Methods

### 2.1. Identification of DE-microRNAs

**Figure 1:**
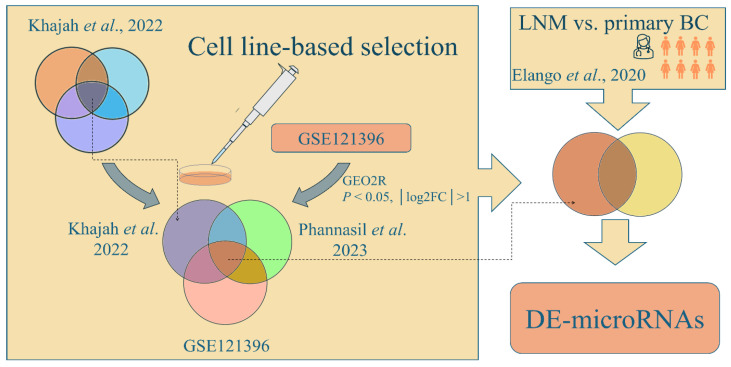
Flow chart of DE-microRNA identification. Selection was based on summarizing results from Khajah et al. [9], Phanassil et al. [10], GSE121396, and Elango et al. [11]. Abbreviations: BC—breast cancer; DE-microRNAs—differentially expressed microRNAs; FC—fold change; and LNM—lymph node metastasis.

**Figure 2:**
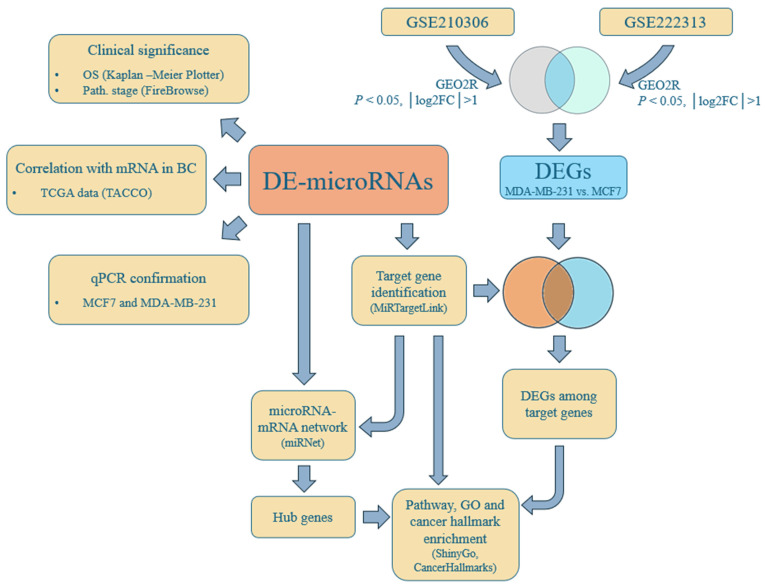
Flow chart of the bioinformatic analysis.

Identification of DE-microRNAs was a multistep procedure, presented schematically in Figure 1. The first step in the cell-line-based selection of DE-microRNAs included the comparison of microRNA expression between BC cell lines with significantly differing invasiveness—less invasive ER-positive MCF7 and highly invasive TNBC cell lines (commonly analyzed MDA-MB-231). For this purpose, lists of the top dysregulated microRNAs in three cell-line comparisons were extracted from the publication by Khajah et al. [9]. These comparisons included TNBC MDA-MB-231 cells and two ER-silenced MCF7 derivatives (pII and IM-26), each compared with an ER-expressing, siRNA-transfected MCF7 derivative (YS1.2). Since datasets from this study were not deposited in GEO or another known open-source database, we extracted three lists of the top 50 DE-microRNAs directly from the paper (Illumina NextSeq500-Illumina, San Diego, CA, USA, filtered using a cutoff of 4 reads, normalization based on the total read count per sample, false discovery rate (FDR) < 0.05). The three lists of DE-microRNAs from Khajah et al. [9], presumably associated with ER-silencing and increased invasiveness, were compared. The overlapping hits were further narrowed down in the microRNA selection process by comparing the results with a DE-microRNA list from another study focusing on MCF7 and MDA-MB-231 cell lines in the context of metastatic potential [10]. In order to further filter out the most plausible candidates, we identified a dataset from the GEO database (GSE121396), which included results from microRNA profiling of MCF7 and triple negative BC cell lines (MDA-MB-231 and MDA-MB-468). The R-based application GEO2R, a web tool which utilizes the GEOquery (v.2.66.0) and limma package (v.3.54.0), was used for the analysis of DE-microRNAs from the corresponding microarray data. The adjusted *p* value < 0.05 and |log_2_fold change| > 1 were used as cutoffs. Common DE-microRNAs from GSE121396 for both comparisons of TNBC vs. MCF7 cells were used in the further selection process. The intersection of the results from all three sources ([9,10] and GSE121396) represented the final set of candidate DE-microRNAs associated with a highly invasive phenotype in BC from a cell line-based approach. In an effort to further augment the significance of the findings and strengthen the indications of the involvement of the selected panel of microRNAs in BC progression, we compared the list of top candidates with DE-microRNAs from an ex vivo study by Elango et al. [11], who profiled microRNAs in primary BC and lymph node metastases (LNM). The data were analyzed as described above. After the identification of DE-microRNAs, the analysis followed the scheme presented in Figure 2.

### 2.2. Clinical Significance of DE-microRNAs

The clinical significance of the identified DE-microRNAs, in terms of overall survival (OS) and the pathological stage of BC, was analyzed using the Kaplan–Meier plotter (http://www.kmplot.com, accessed on 18 December 2025) and FireBrowse 1.1.40. (http://firebrowse.org/, accessed on 18 December 2025) tools, respectively (Figure 2). TCGA data (*n* = 1078, TCGA-BRCA) were used for the OS analysis, with the following parameters: patients were split by the median, automatic selection of the best cutoff in percentiles, no follow-up threshold, and analysis performed with and without restriction according to ER status. The Kruskal–Wallis test was used to assess the association of DE-microRNAs with the pathological stage of BC by employing FireBrowse (*p* value < 0.05 and Q value < 0.3), which also utilizes TCGA data.

### 2.3. Correlation of DE-microRNAs with the Expression of mRNAs in BC

In order to assess the correlation of DE-microRNAs with mRNA expression in invasive BC, we used the Transcriptome Alterations in the CanCer Omnibus (TACCO) database, which relies on TCGA-BRCA data (Figure 2). Log_2_-transformed expression values for microRNAs and mRNAs were used for the correlation analysis, and a Pearson’s correlation coefficient |*r*| > 0.3 was considered an indicator of significant correlation. Bonferroni correction was used for adjusting the corresponding *p* values for multiple comparisons.

### 2.4. Identification of Targets of DE-microRNAs

Sets of mRNAs targeted by DE-microRNAs were identified by searching the miRTargetLink 2.0 (https://ccb-compute.cs.uni-saarland.de/mirtargetlink2, accessed 25 November 2025) database, which integrates data from multiple online resources (miRBase 22.1, miRTarBase 8.0, miRDB 6.0, mirDIP 4.1, miRPathDB 2.0, miRATBase 1.0, miEAA 2.0 and GeneTrail 3). Both predicted and experimentally validated (weak and strong) target hits were included in the subsequent analyses (Figure 2).

### 2.5. Functional Enrichment Analysis: Pathways, Gene Ontology, and Cancer Hallmarks

The online tool ShinyGO 0.85 was used for pathway analysis of the identified DE-microRNA targets. The GO and Kyoto Encyclopedia of Genes and Genomes (KEGG) pathway terms enriched in the sets of microRNA-targeted genes were identified with the FDR cutoff set at 0.05, a minimum pathway size of 2, and a maximum of 5000. Chart diagrams representing the fold enrichment of the GO and KEGG pathway terms, along with the corresponding gene number and −log_10_(FDR), were generated using ShinyGO 0.85. KEGG pathway graphs with highlighted genes from microRNA target lists were generated within the ShinyGO 0.85 platform, using the Pathview R package (v.1.50.0). Target gene lists were uploaded to the online analysis platform available at www.cancerhallmarks.com (accessed on 2 December 2025) [12] for hallmark enrichment analysis, and the integrated cancer hallmark gene set (*n* = 6763) was selected for the comparison (a consensus list of cancer hallmark genes from the available mapping resources) by a hypergeometric test (Figure 2). Our analysis included several iterations of functional enrichment analysis: all predicted/validated targets (selected as described in Section 2.4), network-based prioritization (hub genes from Section 2.6) and an overlap with differentially expressed genes (DEGs) from cell line-based comparison (Section 2.7).

### 2.6. Regulatory and PPI Network Construction

The microRNA–mRNA interaction network was constructed using a microRNA-centric web-based network analysis platform, miRNet 2.0 (https://www.mirnet.ca/, accessed on 27 November 2025) [13]. A list of microRNAs was uploaded, and the analysis parameters were set to *H. sapiens* as the source organism, while miRTarBase 9.0 and TarBase 9.0 were selected as databases for target gene selection. Degree > 300 and betweenness > 50,000 were used to identify the hub genes, which were later subjected to GO and KEGG pathway analysis (Figure 2).

### 2.7. Identification of DEGs

DEGs which encode mRNA from MDA-MB-231 vs. MCF7 comparison were identified by integrating results from two GEO datasets with ≥2 replicates per group—GSE210306 and GSE222313. GEO2R, an R-based web application, was used for the analysis of DEGs, with a *p* value threshold set at 0.05 and |log_2_fold change| > 1. The list of DEGs was compared with the identified microRNA targets (targets of upregulated DE-microRNAs with downregulated DEGs and vice versa) and the overlapping hits were subjected to GO and KEGG pathway analysis, as previously described (Figure 2).

### 2.8. Cell Lines and Cultivation

ER-positive MCF7 cells and triple-negative BC cell lines MDA-MB-231 and MDA-MB-468 were obtained from the American Type Culture Collection (Rockville, MD, USA). Cells were grown in Dulbecco’s Modified Eagle’s Medium (DMEM) (Gibco, Invitrogen, Waltham, MA, USA), supplemented with 10% Fetal Bovine Serum (PAA Laboratories, Pasching, Austria), 100 U/mL penicillin, and 100 μg/mL streptomycin (Capricorn Scientific, Ebsdorfergrund, Germany), under standard culturing conditions, with 5% CO_2_ at 37 °C. After reaching approximately 80% confluency, the cells were detached using trypsin in DPBS (Capricorn Scientific, Germany) and seeded in T25 culture flasks for subsequent RNA isolation. The cells were allowed to adhere and grow under the same standard culture conditions until the required confluency for the experiment was achieved.

### 2.9. Relative Quantification of Putative DE-microRNAs in MCF7, MDA-MB-468 and MDA-MB-231 Cell Lines

Cultured cells lysed in TRIsure reagent (Bioline, Meridian Bioscience, Memphis, TN, USA) (acid guanidinium thiocyanate phenol-chloroform extraction method) were subjected to total RNA isolation by phase separation and alcohol precipitation. The protocol recommended by the manufacturer of the lysis solution (Bioline, Meridian Bioscience, Memphis, TN, USA) was followed, with minor adjustments: to assure the preservation of the RNA’s integrity during isolation and to increase the RNA yield, lysates were thawed on ice and incubation was performed at −20 °C, while RNA precipitation was conducted by using ice-cold isopropanol. The resulting RNA pellet was dissolved in DEPC-treated water and stored at −80 °C, while the yield and purity of the RNA extraction procedure were evaluated spectrophotometrically, based on the absorbance at 260 and 280 nm (Eppendorf BioPhotometer plus, Hamburg, Germany). The DNase treatment (Amplification grade DNase I, Sigma-Aldrich, Burlington, MA, USA) of the extracted RNA, applied to eliminate DNA contamination, was immediately followed by a reverse transcription (RT) reaction, as previously detailed in Stevanović et al. [14]. In brief, the reaction mixture contained 500 ng of the total RNA treated with DNase, 100 U of RevertAid Reverse Transcriptase (Thermo Fisher Scientific, Waltham, MA, USA) and the corresponding Reaction Buffer, 0.5 mM dNTPs and 250 nM stem-loop primers (Microsynth, Balgach, Switzerland) in a volume of 20 µL. The RT reaction mixture was incubated for 30 min at 16 °C, 30 min at 42 °C, and 5 min at 85 °C. Diluted cDNA (30×) was used in the qPCR reaction, which contained SG/ROX qPCR Master Mix (EURx, Gdansk, Poland), primers (Microsynth, Balgach, Switzerland) in equimolar concentrations (0.625 µM) and DEPC-treated water. The temperature profile for the qPCR, performed on the Applied Biosystems 7500 Real-Time PCR System (Thermo Fisher Scientific, MA, USA), was as follows: 10 min at 95 °C, followed by 40 cycles of denaturation at 95 °C for 15 s and primer annealing/elongation at 60 °C for 1 min. *RNU6* was used as an internal reference. The delta-delta Ct (∆∆Ct) method was employed to evaluate the relative expression of microRNAs. Results, presented as fold-changes, were compared using one-way analysis of variance (ANOVA) with a Tukey post hoc test for pairwise comparisons. The primer sequences used for RT and qPCR reactions are given in Table 1.

## 3. Results

### 3.1. Identification of DE-microRNAs

**Figure 3:**
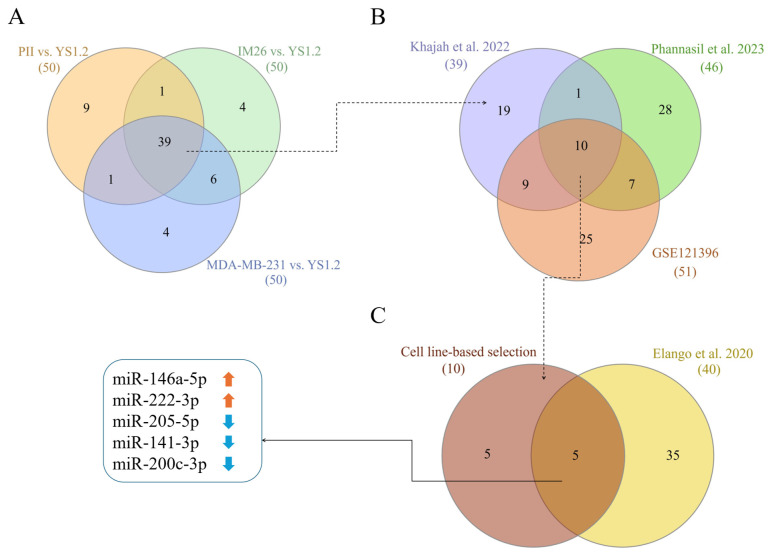
DE-microRNA selection results. Venn diagrams depicting the selection of DE-microRNAs in three different comparisons of microRNA profiles in Khajah et al. [9] (**A**), their intersection with top dysregulated microRNAs from cell line-based studies of Phannasil et al. [10] and GEO dataset GSE121396 (**B**), as well as the resulting matching to the top DE-microRNAs from LNM vs. primary BC comparison (Elango et al. [11]) (**C**). The final panel of DE-microRNAs remaining at the intersection of all analyzed datasets consists of 5 microRNAs (**C**).

Since EMT and increased invasion are both associated with ER silencing, we extracted data from the study by Khajah et al. [9]. This study analyzed dysregulated microRNAs in ER-negative BC cell lines with experimentally silenced ER expression (IM26 and PII originating from shRNA-transfected MCF-7 cells) and in the triple-negative cell line MDA-MB-231, compared to the ER-positive derivative of MCF7 (YS1.2). A total of 39 microRNAs remained in the intersection of top differentially expressed microRNAs from all three comparisons and this set was compared to the dysregulated microRNA pool (*n* = 46) from Phannasil et al. [10], as well as to the DE-microRNA set, which we identified by analyzing data from GSE121396 (common hits for top 30 upregulated and downregulated microRNAs in MDA-MB-231 vs. MCF7 and MDA-MB-468 vs. MCF7), leaving 10 microRNAs at the intersection. One of these (miR-10a-5p) showed contrasting expression changes across different datasets. Further comparison with microRNAs dysregulated in LNM vs. primary BC [11] led to the selection of five candidate DE-microRNAs that were potentially involved in key processes leading to metastasis: miR-146a-5p, miR-222-3p, miR-205-5p and two members of the miR-200 family—miR-141-3p and miR-200c-3p—originating from the same locus (Figure 3).

### 3.2. Clinical Significance of DE-microRNAs

**Figure 4:**
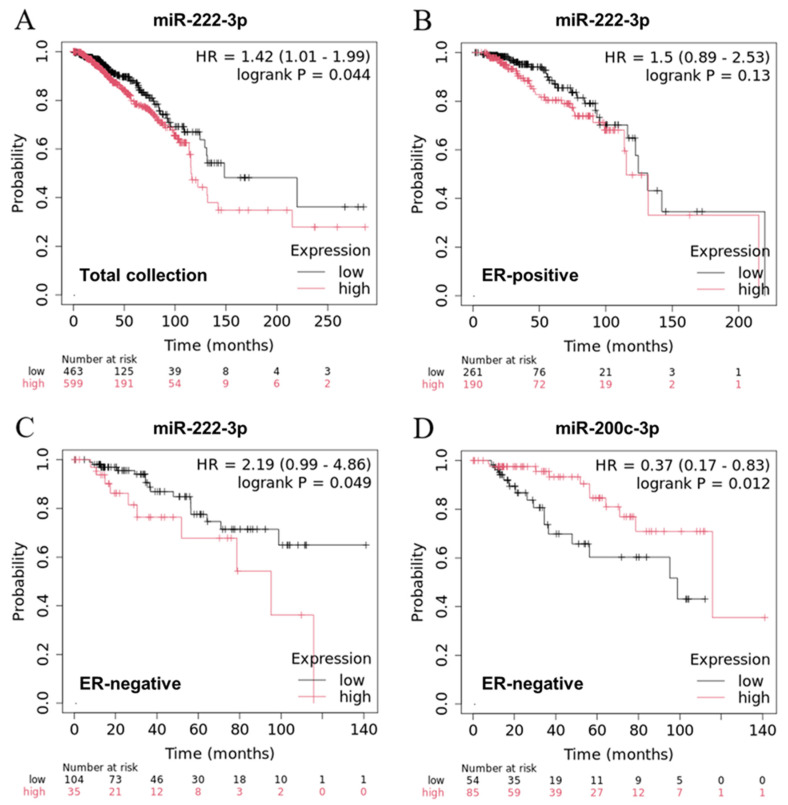
Kaplan–Meier survival curves representing the association between miR-222-3p ((**A**) total collection, (**B**) ER-positive group, (**C**) ER-negative group) and miR-200c-3p expression ((**D**) ER-negative group) with the overall survival in the TCGA-BRCA collection. The hazard ratio (HR) and the corresponding *p* value from the log-rank test are presented within each plot.

Regarding the clinical significance of the putative DE-microRNAs in BC, miR-222-3p demonstrated the association with OS in the TCGA-BRCA collection (Figure 4). Patients with higher expression of this microRNA, which is a known regulator of estrogen signaling, demonstrated poorer OS (HR = 1.42, 95% CI 1.01–1.99), which was accordant with its increased expression in invasive BC and LNM. When patients were stratified according to their ER status, the correlation with OS remained significant in the ER-negative group. A remarkable association with OS was also noted for miR-200c-3p in ER-negative BC, with HR of 0.37 (95% CI 0.17–0.83), which was compatible with its decreased expression in aggressive BC.

MiR-222-3p also demonstrated a significant association with the clinical stage of BC (*p* < 0.001) and was ranked fourth among the top dysregulated microRNAs related to this clinical parameter when ordered by *p* value. Another DE-microRNA hit, miR-205-5p, was also significantly associated with the clinical stage of BC (*p* < 0.001), while the association for miR-141-3p reached only marginal statistical significance (*p* = 0.04).

### 3.3. Correlation of DE-microRNAs with the Expression of mRNAs in BC

According to the integration of microRNA and mRNA profiling results in TCGA-BRCA, the most significant correlation between the expression of our panel of DE-microRNAs and protein-coding genes was detected for miR-146a-5p (*n* = 38), with respect to the number of genes with correlated expression and the corresponding correlation coefficients (Table 2). Genes with both negative and positive correlation coefficients are listed. An examination of the list revealed several obvious cancer-related candidates among the correlated mRNAs, including SHC SH2 domain-binding protein 1 (*SHCBP1*), viperin (*RSAD2*), SERPINE1 mRNA binding protein 1 (*SERBP1*), Notch receptor 1 (*NOTCH1*), Cyclin A2 (*CCNA2*), Toll-like Receptor 2 (*TLR2*), Intercellular Adhesion Molecule 1 (*ICAM1*), C-X-C chemokine receptor type 4 (*CXCR4*), B-cell lymphoma 2-related protein A1 (*BCL2A1*), and RANTES (*CCL5*). These are known promoters of BC EMT, migration, invasion, and metastasis, as well as resistance to proapoptotic stimuli, BC recurrence, and chemoresistance. *ICAM1* also appeared among the mRNAs correlated with miR-222-3p, which, like miR-146a-5p, is overexpressed in BC. As expected, miR-222-3p demonstrated an opposite correlation with the ER expression. For miR-205-5p, none of the correlations were characterized by a correlation coefficient *r* > 0.3. Regarding miR-141-3p and miR-200c-3p, a certain degree of overlap is expected, considering the correlated expression of these two microRNAs transcribed from clustered microRNA genes. The expression of both Zinc Finger E-Box Binding Homeobox 1 and 2 (*ZEB1* and *ZEB2*), which are key regulators of EMT, was inversely correlated with the level of miR-141-3p and miR-200c-3p, consistent with their downregulation in more invasive BC. However, although some of these correlations fitted to the hypothesized functional relations, negative correlations do not necessarily imply that a certain gene is a direct or indirect target of the investigated microRNA. Furthermore, coregulation could originate from the same type of stimuli influencing the expression, including the cell-specific, stage-specific and condition-specific effects. Since we did not correct for technical (batch effect) or biological confounders (tumor heterogeneity, disease stage, etc.), their effect on the detected correlation cannot be excluded.

### 3.4. Pathway, Gene Ontology (GO) and Cancer Hallmark Enrichment Analysis of Genes Targeted by DE-microRNAs

Potential targets of the selected microRNAs were identified as the union of validated and predicted target genes within the MiRTargetLink 2.0 output. After eliminating duplicate results from different tools and databases incorporated within the used web-based platform, the numbers of putative targets were 1442 for miR-146a-5p, 1765 for miR-222-3p, 4575 for miR-205-5p, 2734 for miR-141-3p, and 2528 for miR-200c-3p. As miR-141-3p and miR-200c-3p belong to the same microRNA family, a substantial overlap in their target genes was expected. Our findings confirmed this similarity, as 899 targets were found in both lists.

**Figure 5:**
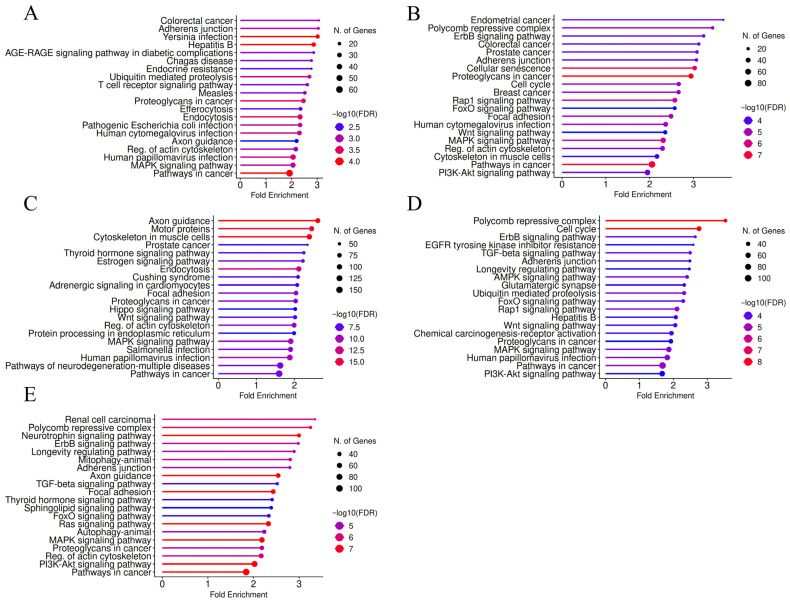
Graphical presentation of KEGG pathway enrichment analysis results for putative target lists of (**A**) miR-146a-5p, (**B**) miR-222-3p, (**C**) miR-205-5p, (**D**) miR-141-3p and (**E**) miR-200c-3p. Terms were ranked according to fold enrichment, while the size of the circles corresponded to the number of genes and the color represented enrichment FDR values.

According to the results of KEGG pathway enrichment analysis, the terms “pathways in cancer”, “adherens junction”, “focal adhesion”, “regulation of actin cytoskeleton”, “proteoglycans in cancer”, “MAPK signaling pathway”, “polycomb repressive complex”, “ErbB signaling pathway”, and “PI3K-Akt signaling pathway” were frequently identified among the top hits for the putative target lists of the selected DE-microRNAs, arranged according to fold enrichment (Figure 5). Supplementary Figure S1 also illustrates the matching in the results of the KEGG pathway enrichment analysis between DE-microRNAs. All of these terms are related to malignant phenotype, proliferation, differentiation, EMT, connection to extracellular matrix, motility, invasion, angiogenesis and metastatic potential. KEGG pathway graphs for “pathways in cancer”, with highlighted microRNA targets, are presented in Supplementary Figures S2–S6. These figures visually illustrate the findings from the KEGG pathway enrichment analysis and demonstrate the connections between specific putative microRNA targets. As seen in Supplementary Figures S2–S6, entire lines of subsequently activated members of certain signaling pathways (MAPK, ErbB, PI3K-Akt) are potentially directly regulated by the same microRNA, which is in line with the potential relevance of DE-microRNAs, in addition to statistical descriptions. Additionally, for miR-222-3p, “breast cancer” was ranked among the top ten enriched terms. According to GO biological process (GO BP) enrichment analysis (Supplementary Figure S7), the enriched terms for all five microRNAs were primarily related to different aspects of transcriptional regulation, biomacromolecules’ metabolism and protein modification. Furthermore, for most of the selected DE-microRNAs, “cell development”, “cell differentiation” and “anatomical structure morphogenesis” were among the most enriched GO BP terms, while motility-related “cytoskeleton organization” and “cell projection organization” were found among the top hits for the putative targets of miR-205-5p.

When the analysis was restricted to experimentally validated targets of DE-microRNAs, the list of genes was markedly reduced (199 for miR-146a-5p, 393 for miR-222-3p, 180 for miR-205-5p, 143 for miR-141-3p, and 212 for miR-200c-3p), and the most obvious reduction concerned mir-205-5p. The reason for this could be a previous research interest in functional properties of this microRNA, compared to other DE-microRNAs. Nevertheless, the results from KEGG pathway and GO BP enrichment analysis remained similar (Supplementary Figures S8 and S9), with cancer-related term repeating in both lists of top hits, including previously mentioned signaling pathways. Downregulated DE-microRNAs shared several top enriched KEGG pathway terms, besides very broad “pathways in cancer” and “MicroRNAs in cancer”, including “endocrine resistance” and terms related to steroid hormone-driven cancers. To note, multiple viral and bacterial infection-related terms, as well as terms associated with immune system-regulating pathways, were enriched within the miR-146a-5p target list, illustrating the known immunoregulatory properties of this microRNA.

**Figure 6:**
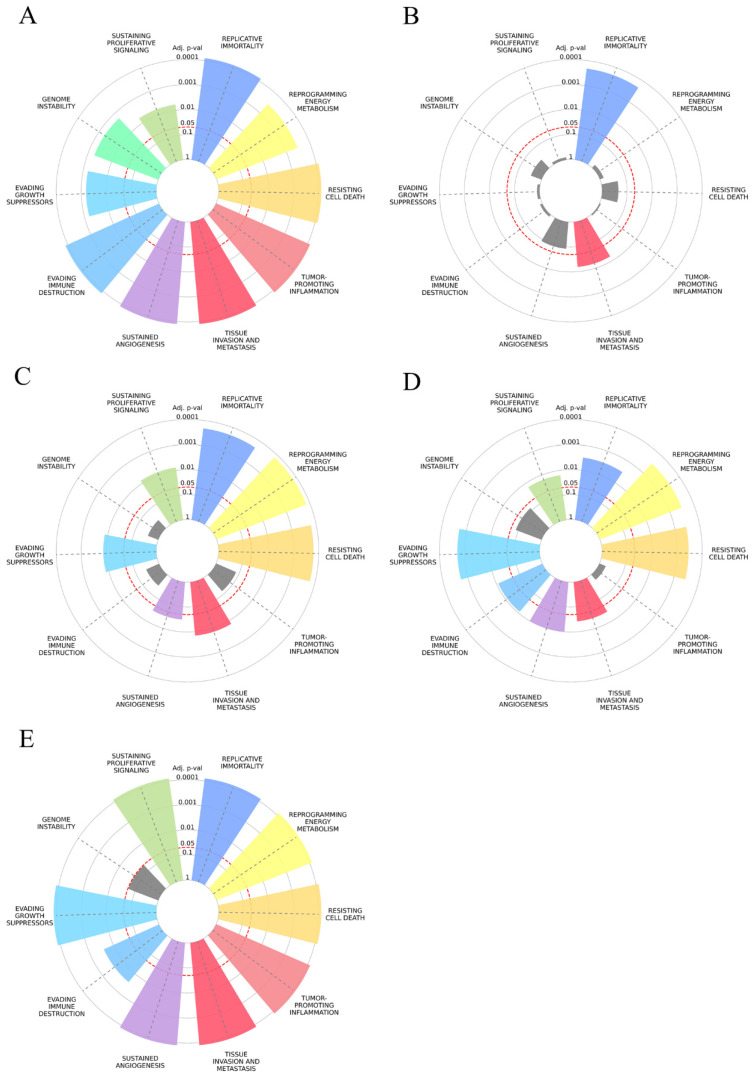
Cancer hallmark enrichment graphs for putative gene target lists of (**A**) miR-146a-5p, (**B**) miR-222-3p, (**C**) miR-205-5p, (**D**) miR-141-3p and (**E**) miR-200c-3p. Hallmarks with no significant enrichment are presented in gray. *p* values are indicated by the distance from the center of the circle, with the red dotted line representing a 0.05 threshold.

Regarding the cancer hallmark enrichment, the number of input results was too large for the analysis, so we focused on validated microRNA targets. According to the results (Figure 6), genes associated with cancer hallmarks, such as “resisting cell death” or “replicative immortality”, were highly overrepresented within the target lists of the selected DE-microRNA. Regarding “tissue invasion and metastasis”, all target gene sets of selected microRNAs demonstrated enrichment in genes associated with this hallmark. For miR-200c-3p, this was the top enriched hallmark, with the adjusted *p* value of 7.8 × 10^−15^ and an odds ratio (OR) of 3.5 from the enrichment analysis.

### 3.5. Regulatory microRNA–mRNA Network and Hub Genes

**Figure 7:**
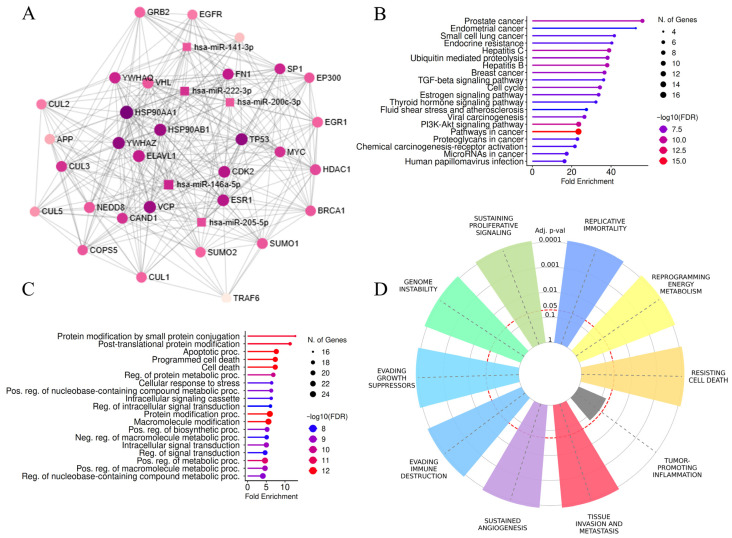
Analysis of hub genes from DE-microRNA–mRNA network constructed via miRNet 2.0. (**A**) DE-microRNA-hub gene network—the color of the nodes represents the degree of interaction, with darker shades corresponding to higher degrees; (**B**) graphical presentation of KEGG pathway enrichment analysis results for hub genes; (**C**) graphical presentation of GO BP enrichment analysis results for hub genes; and (**D**) cancer hallmark enrichment graphs for hub genes, with red circle representing statistical significance threshold.

Using the miRNet 2.0 platform, we constructed a regulatory network incorporating all five DE-microRNAs and PPI, which was evaluated for topology parameters, leading to the identification of the 31 hub genes presented in Figure 7. Even when separate networks were constructed for upregulated and downregulated microRNAs, the number of matching genes among hubs was significant among groups (10/15 and 10/17, respectively). Hub genes were enriched in pathways related to malignant diseases, including “breast cancer”, “pathways in cancer”, “microRNAs in cancer”, “TGF-beta signaling pathway”, “estrogen signaling pathway”, “PI3K-Akt signaling pathway”, and “proteoglycans in cancer”, which may be related to an invasive BC phenotype (Figure 7B). When biological processes were considered, terms related to cell death and protein modifications showed the highest fold enrichment (Figure 7C). Halmark enrichment analysis further supported a significance of selected DE-microRNAs for acquiring an invasive BC phenotype, since “tissue invasion and metastasis” was one of the three most enriched cancer hallmarks (the other two were “sustained angiogenesis” and “resisting cell death”), with an adjusted *p* value < 10^−6^ and an OR of 7.14 (Figure 7D).

### 3.6. Analysis of DEGs from MDA-MB-231 vs. MCF7 Comparisons

Besides analyzing the whole putative target lists of DE-microRNAs and the supposed hub genes, we used an approach aimed at identifying DEGs in mRNA profiling studies involving relevant cell lines and comparing them to target lists. By identifying DEGs from two different datasets (GSE210306 and GSE222313) and combining the results, we determined the intersections with a putative target list corresponding to each microRNA, while taking into account the direction of the change in the expression (opposite for microRNA and DEGs).

**Figure 8:**
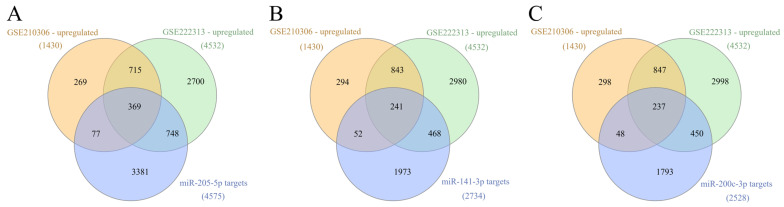
Venn diagrams depicting the intersection of upregulated DEGs from MDA-MB-231 vs. MCF7 comparison (GEO datasets GSE210306 and GSE222313) with putative targets of miR-205-5p (**A**), miR-141-3p (**B**) and miR-200c-3p (**C**).

**Figure 9:**
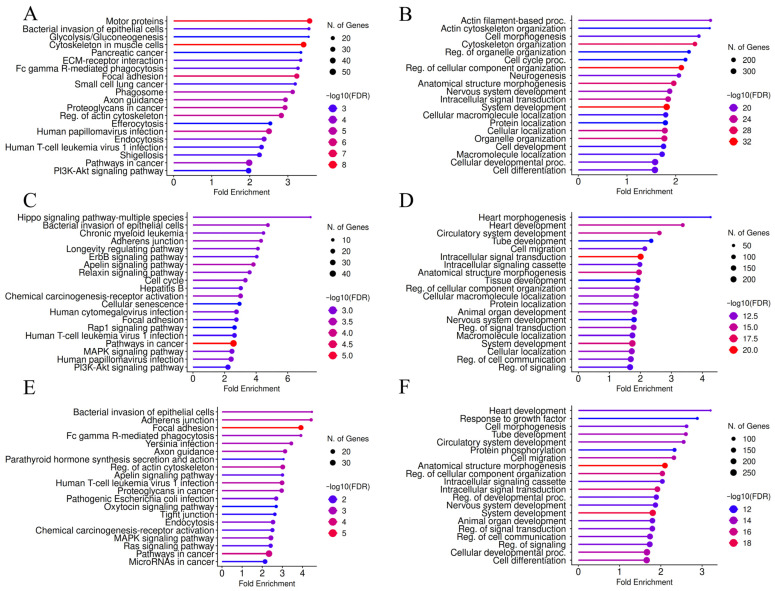
Graphical presentation of KEGG pathway and GO BP enrichment analysis results for upregulated DEGs among putative targets of downregulated DE-microRNAs. (**A**) KEGG pathway analysis and (**B**) GO BP enrichment analysis corresponding to miR-205-5p; (**C**) KEGG pathway analysis and (**D**) GO BP enrichment analysis corresponding to miR-141-3p; (**E**) KEGG pathway analysis and (**F**) GO BP enrichment analysis corresponding to miR-200c-3p. Terms were ranked according to fold enrichment, while the size of the circles corresponded to the number of genes and the color represented enrichment FDR values.

**Figure 10:**
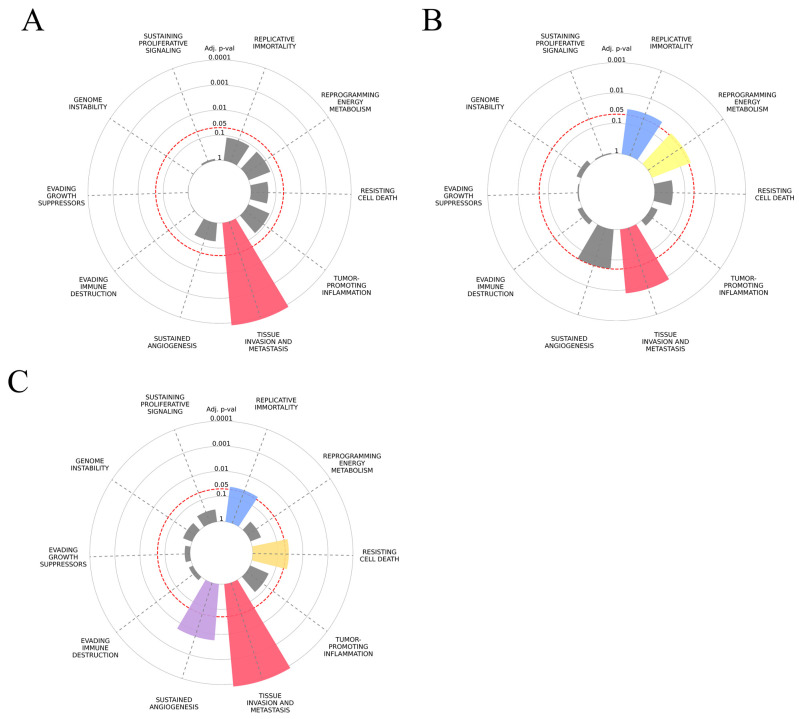
Cancer hallmark enrichment graphs for upregulated DEGs from MDA-MB-231 vs. MCF7 comparison (GEO datasets GSE210306 and GSE222313) within putative target lists of miR-205-5p (**A**), miR-141-3p (**B**) and miR-200c-3p (**C**). Red circles represent statistical significance threshold.

When it comes to miR-146a-5p, the KEGG pathway term “choline metabolism in cancer” was the only one enriched in the targeted genes downregulated in the TNBC cell line (*n* = 273) (Supplementary Figure S10), while none of the cancer hallmarks demonstrated significant enrichment. This result illustrated that stringent conditions during the selection of putative targets as entries in the functional enrichment analysis result in the poor association of miR-146a-5p with functional terms that are relevant for BC pathogenesis. Additionally, this specific enriched pathway term is biologically related to MAPK signaling found in previous iterations of pathway enrichment analysis, which included all putative miR-146a-5p targets. However, choline metabolism is associated with proliferation and cell survival, rather than with invasiveness. For miR-222-3p, on the other hand, targeted genes among DEGs (*n* = 335) were enriched in KEGG pathway terms related to breast cancer ER status, invasion and metastasis, such as “ErbB signaling pathway”, “endocrine resistance”, “prolactin signaling pathway”, “breast cancer”, “proteoglycans in cancer”, “PI3K-Akt signaling pathway” and “pathways in cancer”, which further supports the hypothesized role of this DE-microRNA (Supplementary Figure S11). However, hallmark enrichment analysis demonstrated significant results for “replicative immortality”, which may be related to a relatively small number of genes included in the analysis. Furthermore, the stringent conditions for the selection of putatively targeted genes may have affected the results. On the other hand, upregulated DEGs present within lists of targets of downregulated DE-microRNAs (mir-205-5p, miR-141-3p and miR-200c-3p) (Figure 8) showed enrichment in multiple invasive BC-related KEGG pathway and GO BP terms (Figure 9, Supplementary Figures S12 and S13), with the most significant cancer hallmark enrichment being for “invasion and metastasis” (Figure 10). Additionally, all four genes with inversely correlated expression with miR-141-3p in BC (from TACCO database analysis) were present in the analyzed set of DEGs corresponding to this microRNA. In the case of miR-200c-3p, similar results were found, with a large percentage (66%) of genes being present in both lists.

### 3.7. Relative Expression of DE-microRNAs in MCF7, MDA-MB-468 and MDA-MB-231 Cells

**Figure 11:**
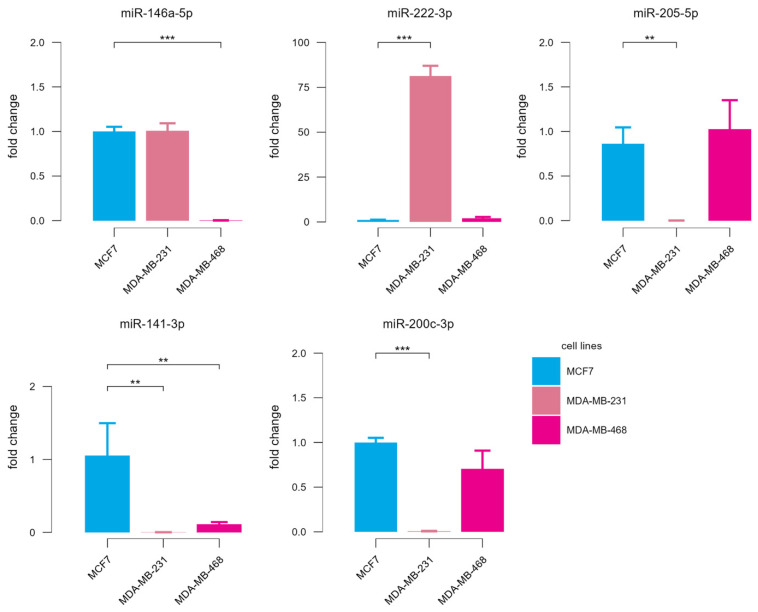
Relative expression of putative DE-microRNAs in MCF7 and TNBC cell lines MDA-MB-231 and MDA-MB-468. Bar plots represent mean fold changes, relative to MCF7 cells + SD; *p* < 0.01 is indicated by two asterisks and *p* < 0.001 by three asterisks.

In order to confirm the results of high-throughput analyses and to provide additional experimental evidence of the differential expression of putative DE-microRNAs, we conducted RT-qPCR analysis of RNAs isolated from relevant cell lines (Figure 11). As expected, highly invasive MDA-MB-231 cells demonstrated much larger difference in the expression of putative DE-microRNAs in relation to MCF7, compared to MDA-MB-468. Except for miR-146a-5p, all other selected microRNAs showed the direction of change in the expression in invasive BC cells corresponding to previous microRNA profiling results used in the DE-microRNA selection process. For miR-222-3p, an increase in the expression followed a trend from MCF7, over MDA-MB-468, to MDA-MB-231, although a post hoc Tukey test did not show statistical significance for the pairwise comparison of MDA-MB-468 vs. MCF7. Similarly, mean expression levels of miR-141-3p and miR-200c-3p demonstrated a consistent decrease from MCF7 to MDA-231, even though statistical significance was not reached for miR-200c-3p in MDA-MB-468 vs. MCF7 comparison (*p* = 0.055).

## 4. Discussion

Dysregulation of microRNA expression in BC is well documented and has been associated with molecular disturbances involved in key pathways associated with cancer initiation, progression and metastasis. Suppression of the expression or an upregulation of various microRNAs was found to contribute to EMT, cell motility, invasion, anchorage independence and related processes involved in BC metastasis [15]. Additionally, specific microRNAs act as endocrine modulators in BC, thereby influencing the biological behavior of the tumor, progression of the disease and anti-hormonal drug responses [16]. Due to these functional properties, tissue- and cellular state-specific expression patterns and context-dependent activities, microRNAs were recognized as promising novel theranostic tools that are urgently needed to overcome challenges in BC clinical diagnosis, prognosis, treatment and recurrence monitoring [17].

The objective of the current study was to employ bioinformatics tools to identify and characterize a set of microRNAs acting as plausible candidates for key players involved in EMT, migration, invasion and/or hormonal regulation in BC. Identification of such candidates, or a metastatic BC microRNA signature, would represent a valuable starting point for experimental validation. Also, such a discovery would be invaluable for determining specific microRNA–mRNA networks and microRNA-controlled pathways which significantly contribute to acquiring aggressive malignant phenotype, novel biomarker search and therapeutic target evaluation. We systematically integrated microRNA profiling data from three different studies (by Khajah et al. [9], Phannasil et al. [10] and Oltra et al. [18]—GSE121396) on BC cell lines with different invasive capabilities. Additionally, we compared the microRNA set from the intersection of these studies with differentially expressed microRNAs between LNM and primary BC [11], which resulted in five matching microRNA hits—DE-microRNAs miR-146a-5p, miR-222-3p, miR-205-5p, miR-141-3p and miR-200c-3p. This set of supposed DE-microRNAs was subjected to further evaluation of their clinical significance in BC, target prediction, microRNA–mRNA network construction, and functional enrichment analysis. Functional enrichment analysis included several iterations: one without prioritization and others with filtering of microRNA targets/hub genes based on experimental validation, network parameters and mRNA expression in BC. We used this approach to illustrate that regardless of the prioritization approach used, selected DE-microRNAs remain associated with cancer aggressiveness-related terms and, moreover, that a stringent approach further filters out targets related to invasiveness and metastasis, compared to other cancer features and hallmarks.

The results of DE-microRNA identification are not surprising, since various studies suggested the involvement of these five microRNAs in different aspects of BC pathogenesis. Namely, miR-222-3p is a known oncogenic microRNA, especially in steroid hormone-driven cancers, which is in line with our finding of its upregulation in invasive BC cell lines, TNBC and metastatic cancer [19,20]. Besides repetitive upregulation in the datasets used for DE-microRNA selection, this microRNA also demonstrated the most prominent dysregulation in our qPCR experiment evaluating BC cell lines. Previous experimental stimulation and silencing in relevant BC cell lines also demonstrated a significant impact on cell invasion, as well as a pro-oncogenic effect in the tumor microenvironment [20,21,22,23].

Based on the premise that microRNAs associated with metastatic potential in BC could be relevant for clinical prognosis and cancer characteristics, we evaluated the correlation between the expression of the proposed DE-microRNAs and the overall survival and clinical stage in BC, based on the data from TCGA-BRCA. Taking into account the relationship between miR-222-3p and estrogen signaling [24], as well as the strong experimental evidence supporting the importance of this microRNA for BC pathogenesis, the detected negative correlation of its expression with the overall survival in BC patients and the dependency on ER status are concordant with the hypothesized role of miR-222-3p in BC progression. Additionally, we found that the level of expression of miR-222-3p is associated with the clinical stage of BC, which is another indication of potential clinical significance and the involvement in BC progression. These findings are in line with the results of clinical studies demonstrating increased levels of miR-222-3p in tumor tissues from patients with metastases and TNBC, as well as with findings suggesting higher levels of circulatory miR-222-3p in patients with a higher BC stage and shorter disease-free survival [25,26,27].

When we analyzed the correlation between the expression of miR-222-3p and mRNA levels in tumor samples, a negative relation with *ESR1* was detected, in line with the results of target prediction and experimental validation [21,24]. Among other genes with correlated expression, some are previously experimentally validated targets of miR-222-3p [28,29,30,31,32], among which *ICAM1* stands out as being strongly associated with BC invasiveness and metastatic potential [33,34,35]. However, since genes with correlated expression in BC tissue may not be direct or indirect targets of microRNA regulation, we focused our GO, pathway and cancer hallmark enrichment analysis on predicted/validated target genes. For miR-222-3p, some of the top enriched pathways corresponded to different types of cancer, including BC, suggesting a common pro-oncogenic effect of this microRNA, which aligns with the data from the literature [36]. Other enriched pathway terms are in line with the proposed role in EMT, motility, invasion and metastatic potential, including an experimentally confirmed relation to Wnt/β-catenin signaling [20]. Furthermore, cancer hallmark enrichment analysis supported the potential role of miR-222-3p in metastasis, since the “tissue invasion and metastasis” term was one of the only two enriched hallmarks, linking this feature with miR-222-3p upregulation, although causal relations cannot be directly inferred from in silico analysis. Another approach in investigating the potential functional properties of DE-microRNAs was to crosscheck the lists of target genes with DEGs from the comparison of TNBC and low-invasive BC cell lines. When this subset of DEGs among miR-222-3p targets was subjected to the same analysis, KEGG pathways related to estrogen receptor signaling in breast cancer, invasion and metastasis remained the top enriched terms, while for GO BP enrichment analysis, the top hits were related to cell adhesion. All these results qualify miR-222-3p for one of the most plausible candidates for oncogenic DE-microRNAs with significant roles in BC metastasis, and warrant further experimental evaluations.

Besides miR-222-3p, another microRNA—miR-146a-5p—was selected as an upregulated DE-microRNA in the present analysis. Unlike miR-222-3p, this microRNA demonstrated quite ambiguous functions in previous experimental studies on BC and other malignancies. More important to the present study is that our microRNA quantification results were also inconsistent with the proposed role of miR-146a-5p as an upregulated DE-microRNA in TNBC cell lines. Namely, even though profiling results from studies and the corresponding datasets included in our DE-microRNA selection were in agreement and suggested that miR-146a-5p is upregulated in highly invasive BC cells, conflicting results were found in several studies [37,38,39,40,41] and in our qPCR experiment on the same cell lines. Therefore, cell line-specific effects are not supported, although BC subtype-related specificities cannot be excluded. One of the explanations for discrepancies in miR-146a-5p quantification is the strong effect of culturing conditions, handling, methodological approaches and intrinsic biological differences. On the other hand, multiple mechanistic studies confirmed the pro-oncogenic properties of miR-146a-5p in BC [42,43,44,45,46], which may indicate a potentially complex, context-dependent dual role of this microRNA in BC. Besides specific activities in malignant cells, the cancer-related functions of this microRNA are also attributed to its known role as an immune system regulator [47] and changes in its expression in cancerous tissue may relate to immune infiltration and changes in tumor microenvironment as well. Since we focused on pro-oncogenic effects, due to a detected upregulation in invasive cell lines and metastatic BC from microRNA profiling studies, the effects of other potential miR-146a-5p targets, which are upregulated in invasive BC, may be underestimated. However, the results from our qPCR experiment and other candidates for microRNA-based studies did not support the upregulation and our selection of miR-146a-5p as one of the top candidates for DE-microRNAs. Other iterations of functional enrichment analysis unrelated to the direction of changes in the expression of miR-146a-5p and its putative targets still point to the potential cancer-related properties of this microRNA, while BC tissue-derived data showed both tumor suppressors and proto-oncogenes within the list of genes with correlated expression. Specifically, versatile role of miR-146a-5p in BC is also illustrated by the number and the characteristics of genes with correlated expression in BC tissues from TCGA-BRCA. Among the listed genes (*n* = 38), most were positively correlated with miR-146a-5p expression and described as promoters of BC invasiveness. Therefore, the expression of these genes may be indirectly regulated by miR-146a-5p, or the expression of microRNA and mRNAs could be unrelated, yet influenced by the same type of stimuli. Furthermore, miR-146a-5p and mRNAs from the list could be involved in negative feedback loops in which the upregulation of one molecule induces the compensatory response of its regulator. Besides known oncogenes, tumor suppressors or genes with a dual role in BC, such as *ERBB4*, *RARB*, *STAT1* and *TRIM22* [48,49,50,51], are also listed as genes with correlated expression with miR-146a-5p, which supports its versatile function and potential complex behavior in BC. Additionally, beside cell-type-specific, this microRNA may express stage-specific effects during BC initiation and progression, which further complicates its qualification as pro-oncogenic and pro-metastatic microRNA.

Cancer hallmark and pathway enrichment analysis on miR-146a-5p targets supported their potential involvement in various aspects of carcinogenesis and metastasis, while infection-related terms and “T cell receptor signaling pathway” were also among the top hits, highlighting the known role of this microRNA in immune system regulation. “Endocrine resistance” also emerged as one of the top enriched terms, supporting the possible involvement of miR-146a-5p in BC progression and modulation of anti-hormonal drug responses. However, this feature could be associated with the selection criteria used for DE-microRNAs, since we based cell line-oriented approach on differential expression in ER+ and ER- cells. Focusing on DEGs among putative miR-146a-5p targets yielded poor results in cancer hallmark and pathway enrichment analysis, probably due to the small number of included genes.

Other three microRNAs (miR-205-5p, miR-141-3p and miR-200c-3p) in DE-microRNA set were consistently downregulated in more aggressive BC cells, according to our present results from the in silico analysis, and previous experimental findings from BC cell lines and clinical studies. Our microRNA quantification results also supported a severe downregulation of all three microRNAs in highly invasive MDA-MB-231 cells, while for MDA-MB-468 with lower metastatic potential, a weaker reduction in the expression was noted. As in miR-200c-3p and miR-141-3p, which belong to the same microRNA family, the similarity in findings is probably related to their structural homology and transcriptional coregulation. All three downregulated DE-microRNAs are well recognized tumor-suppressive microRNAs in BC with significant biomarker potential [52], which matches our findings of their downregulation associated with more invasive BC, TNBC subtype and metastases [53,54]. Bearing all this in mind, our findings, which suggest a strong correlation between the BC tissue expression of miR-200c-3p and the overall survival in aggressive ER-negative BC patients, are aligned with the high relevance of this microRNA for BC progression and response to therapy. Regarding our findings from the other two downregulated DE-microRNAs, their association with the tumor stage in the TCGA-BRCA dataset supports their potential involvement in molecular and cellular mechanisms underlying BC progression. These results originating from a large heterogeneous cohort are prone to bias, due to batch effects and other biological and technical confounders. However, quantification in other clinical samples from various smaller studies corroborated the anti-tumor role of these microRNAs in BC and the association of their downregulation with BC progression, negative treatment outcomes and higher stages [52,54,55]. Experimental silencing and/or induction of expression revealed the crucial role of miR-200c-3p, miR-141-3p and miR-205-5p in EMT, migration and invasion [53,54,55,56,57,58,59,60,61,62,63,64,65,66,67,68,69,70,71,72,73,74]. The main mechanism responsible for their vital role in EMT relies mostly on targeting transcriptional repressors of E-cadherin, namely ZEB1 and ZEB2 [56,57,61,70]. Therefore, it is not surprising that the expression of *ZEB1* and *ZEB2* is listed as being inversely correlated with the levels of tumor-suppressive DE-microRNAs in BC, according to our findings from the analysis of TCGA-BRCA data. Even though the detected correlations do not directly imply a functional relation and inhibitory effects of miR-200c-3p, miR-141-3p and miR-205-5p on the expression of *ZEB1* and *ZEB2*, the direction of correlation corresponds to hypothesized features of these microRNAs. Additionally, TCGA data on mRNA expression was compatible with mRNA profiling results in TNBC and less aggressive BC cell lines, and genes with inversely correlated expression with miR-141-3p (*ZEB1*, *ZEB2*, *ZFPM2* and *RAB8B*) were present within DEGs list and matched with validated miR-141-3p targets identified via MiRTargetLink. It is also worth noting that there was a significant overlap between the genes with inversely correlated expression compared with miR-200c-3p in BC tissue (TCGA data) and a list of DEGs among miR-200c-3p targets. Therefore, prioritization according to all criteria used would be expected to result in similar findings regarding subsequent functional enrichment analysis. All these results point to miR-200 family members as potential drivers of the protective microRNA-based mechanisms in BC progression and metastasis.

Analysis of the potential functional properties of putative targets of miR-200c-3p, miR-141-3p and miR-205-5p further augmented the indication of the supposed relevance of these microRNA for BC progression and aggressiveness, since results from the pathway enrichment analysis corresponded to EMT, cell motility, invasion and metastasis processes. Additionally, functional analysis of putative miR-205-5p targets showed significant gene enrichment for the “estrogen signaling pathway”, which is crucially relevant for BC pathogenesis and invasiveness. Under more stringent conditions for target gene selection, when the analysis was restricted to TNBC DEGs among the target gene list, high enrichment was again detected for various aggressive BC-related KEGG pathways and GO BP terms. The literature data corresponded to these findings, since, for instance, putative targets of miR-141-3p were enrichment in the KEGG terms “Wnt signaling pathway”, “MAPK signaling pathway” and “EGFR tyrosine kinase inhibitor resistance”, which is consistent with experimentally confirmed involvement in Wnt/β-catenin and EGFR signaling in BC [63,64]. With a large validated target list included in hallmark enrichment analysis, “tissue invasion and metastasis” was the main enriched term in cancer for miR-200c-3p, while prioritization according to DEGs among putative targets resulted in even larger extent of filtering genes associated with this hallmark, which remained the only or the top hit for all three microRNAs. Therefore, using more stringent criteria for target selection led to further narrowing of the enriched terms to metastasis and invasiveness-related subgroups. Considering these results and the clinical significance of miR-200c-3p and mir-141-3p, both microRNAs from the cluster demonstrate potential relevance for BC aggressiveness and metastatic potential, with miR-200c-3p showing more pronounced association with an invasive BC phenotype. Similarly, these findings supported the hypothesized role of miR-205-5p in BC progression, and highlighted invasion and metastasis as top candidate processes that are potentially regulated by this microRNA in BC.

To identify a potentially crucial set of genes regulated by putative DE-microRNAs, we constructed a large microRNA–mRNA network broadened with the PPI network and discovered 30 hub genes, most of which appeared in a hub genes list, even when we separated networks for upregulated and downregulated microRNAs. This concordance, as well as the results of the KEGG pathway, GO BP and cancer hallmark enrichment analysis on the hub genes, further supported the supposed role of selected DE-microRNAs in metastasis-related processes in BC, as well as in other features of aggressive malignancies.

Taking into account that the present analysis of potential functions of selected microRNAs in BC pathogenesis was mostly conducted in silico, experimental studies and clinical data are necessary for confirming the obtained results. As in any bioinformatics study, the present analysis provided results and conclusions which are hypothesis-generating, rather than evidence of causal relations, due to inherent limitations. However, focusing on a limited set of gene regulators with the proposed key roles in obtaining an aggressive malignant phenotype in BC has a potential for biomarker discovery outcome prediction, decision-making and treatment monitoring, as well as for enhancing the therapeutic effectiveness.

## 5. Conclusions

In an attempt to identify and functionally characterize microRNAs acting as potential relevant players in BC EMT, migration, invasion, hormonal regulation and metastasis, we employed bioinformatics tools to systematically integrate microRNA profiling data from BC cell lines and from LNM vs. primary BC comparison, which resulted in five top hits—DE-microRNAs miR-146a-5p, miR-222-3p, miR-205-5p, miR-141-3p and miR-200c-3p. According to our findings, among upregulated DE-microRNAs, miR-222-3p displayed a distinctive association with pro-metastatic features, supported by the evaluation of its clinical relevance, expression in BC cell lines, and the results of the GO BP, pathway and cancer hallmark enrichment analysis of target genes. However, in silico findings may only be hypothesis-generating, and not evidence of causal relation. On the other hand, results on miR-146a-5p demonstrated its ambiguous function in terms of BC pathogenesis. Downregulated DE-microRNAs, members of the miR-200 family and miR-205-5p showed a significant association between the suppression of expression and negative clinical features, such as OS and a high clinical stage of BC. These microRNAs also exhibited significant enrichment of genes related to migration, invasion and metastasis among their predicted targets. The obtained results are in line with the potential functional relevance of selected DE-microRNAs in BC, except for miR-146a-5p, which probably performs a more complex role in determining the malignant potential of BC cells. Bearing in mind the primarily in silico nature of the present study and the inherent limitations of bioinformatics evaluation, results need to be taken with caution, discussed with a critical appraisal and considered to be indicative, rather than validated and definitive.

## Figures and Tables

**Table 1:** Primer sequences.

	Primer	Sequence
RT-PCR	RNU6-RT	5′-GTCGTATCCAGTGCAGGGTCCGAGGTATTCGCACTGGATACGACAAAAATATGG-3′
	miR-146a-5p-RT	5′-GTCGTATCCAGTGCAGGGTCCGAGGTATTCGCACTGGATACGACAACCCA-3′
	miR-222-3p-RT	5′-GTCGTATCCAGTGCAGGGTCCGAGGTATTCGCACTGGATACGACACCCAG-3′
	miR-141-3p-RT	5′-GTCGTATCCAGTGCAGGGTCCGAGGTATTCGCACTGGATACGACCCATCT-3′
	miR-200c-3p-RT	5′-GTCGTATCCAGTGCAGGGTCCGAGGTATTCGCACTGGATACGACTCCATC-3′
	miR-205-5p-RT	5′-GTCGTATCCAGTGCAGGGTCCGAGGTATTCGCACTGGATACGACCAGACT-3′
qPCR	Universal miR-rv	5′-CCAGTGCAGGGTCCGAGGTAT-3′
	RNU6-fw	5′-GCGGTCGCAAGGATGACACG-3′
	miR-146a-5p-fw	5′-CGGCGGTTGAGAACTGAATTCCA-3′
	miR-222-3p-fw	5′-CGGCGGTAGCTACATCTGGCTA-3′
	miR-141-3p-fw	5′-CGGCGGTTAACACTGTCTGGTAA-3′
	miR-200c-3p-fw	5′-GGCTGGCTAATACUGCCGGGTAAT-3′
	miR-205-5p-fw	5′-TCGGCUCCUUCAUUCCACCGG-3′

**Table 2:** Correlation between the expression of DE-microRNAs and mRNAs in BC samples from TCGA-BRCA.

miR-146a-5p		miR-222-3p		miR-141-3p		miR-200c-3p	
Gene	*r*	Gene	*r*	Gene	*r*	Gene	*r*
*LFNG*	−0.325	*TSPAN13*	−0.39	*ZEB1*	−0.421	*TIMP2*	−0.435
*ERBB4*	−0.315	*TLE3*	−0.372	*ZEB2*	−0.411	*CDH11*	−0.394
*ZNF629*	−0.31	*MIDN*	−0.353	*ZFPM2*	−0.344	*ZEB2*	−0.394
*CPM*	0.301	*PIGQ*	−0.327	*RAB8B*	−0.316	*ADAM12*	−0.393
*SHCBP1*	0.305	*TCEAL1*	−0.31	*PHB2*	0.324	*TMEM119*	−0.384
*GPM6B*	0.307	*ESR1*	−0.304			*EDNRA*	−0.383
*RSAD2*	0.308	*ZBTB7A*	−0.301			*FOXO1*	−0.38
*RARB*	0.309	*PPP1R14C*	0.317			*ZEB1*	−0.379
*IFIT3*	0.313	*ICAM1*	0.325			*ZFHX4*	−0.374
*SERBP1*	0.318	*CDK6*	0.354			*SHOX2*	−0.36
*NOTCH1*	0.322	*SOD2*	0.365			*JAZF1*	−0.357
*SFRP1*	0.33	*ENO1*	0.374			*IGF2*	−0.347
*OASL*	0.345	*LYN*	0.407			*GPX8*	−0.345
*CCNA2*	0.346					*OSTM1*	−0.344
*FAS*	0.353					*WIPF1*	−0.338
*IFI44L*	0.358					*SEC23A*	−0.326
*IFI44*	0.37					*FN1*	−0.324
*SAMD9L*	0.387					*ZFPM2*	−0.322
*TLR2*	0.4					*DLC1*	−0.321
*ICAM1*	0.403					*LPAR1*	−0.309
*KDM2B*	0.416					*PTPRD*	−0.308
*MX2*	0.426						
*NMI*	0.431						
*BTN2A2*	0.437						
*CXCR4*	0.438						
*CD80*	0.44						
*STAT1*	0.441						
*TRIM22*	0.443						
*LBR*	0.462						
*EPSTI1*	0.469						
*GIMAP4*	0.469						
*LIMD2*	0.484						
*ITGB2*	0.497						
*CD40LG*	0.51						
*CYTIP*	0.513						
*GRAP2*	0.529						
*BCL2A1*	0.623						
*CCL5*	0.638						

*r*—Pearson’s correlation coefficient.

## Data Availability

This article used data from publicly available datasets within the GEO database (GSE121396, GSE210306 and GSE222313). Other relevant data supporting the conclusions of this article are presented within the article or Supplementary Materials.

## Supplementary Materials

The following supporting information can be downloaded at: https://www.mdpi.com/article/10.3390/biology15080617/s1. Figure S1: Sankey plot depicting the results of the KEGG pathway enrichment analysis results (top 20 enriched terms) for putative target lists of miR-146a-5p, miR-222-3p, miR-205-5p, miR-141-3p and miR-200c-3p. Figure S2: KEGG pathway graph for “pathways in cancer” term with putative targets of miR-146a-5p highlighted in red; Figure S3: KEGG pathway graph for “pathways in cancer” term with putative targets of miR-222-3p highlighted in red; Figure S4: KEGG pathway graph for “pathways in cancer” term with putative targets of miR-205-5p highlighted in red; Figure S5: KEGG pathway graph for “pathways in cancer” term with putative targets of miR-141-3p highlighted in red; Figure S6: KEGG pathway graph for “pathways in cancer” term with putative targets of miR-200c-3p highlighted in red; Figure S7: Graphical representation of GO BP enrichment analysis results for putative target lists of (A) miR-146a-5p, (B) miR-222-3p, (C) miR-205-5p, (D) miR-141-3p and (E) miR-200c-3p; Figure S8: Graphical representation of KEGG pathway enrichment analysis results for experimentally validated targets of (A) miR-146a-5p, (B) miR-222-3p, (C) miR-205-5p, (D) miR-141-3p and (E) miR-200c-3p; Figure S9: Graphical representation of GO BP enrichment analysis results for experimentally validated targets of (A) miR-146a-5p, (B) miR-222-3p, (C) miR-205-5p, (D) miR-141-3p and (E) miR-200c-3p; Figure S10: Venn diagram depicting the intersection of upregulated DEGs from MDA-MB-231 vs. MCF7 comparison (GEO datasets GSE210306 and GSE222313) with putative targets of miR-146a-5p; Figure S11: Analysis of DEGs present within target list corresponding to miR-222-3p; Figure S12: Sankey plot depicting the results of the KEGG pathway enrichment analysis results (top 20 enriched terms) for DEGs among putative targets of miR-205-5p, miR-141-3p and miR-200c-3p; Figure S13: Sankey plot depicting the results of the GO BP enrichment analysis results (top 20 enriched terms) for DEGs among putative targets of miR-205-5p, miR-141-3p and miR-200c-3p.

## Abbreviations

The following abbreviations are used in this manuscript:

BC	Breast cancer
DE-microRNAs	Differentially expressed microRNAs
TNBC	Triple negative breast cancer
HER2	Human Epidermal Growth Factor Receptor 2
EMT	Epithelial–mesenchymal transition
ER	Estrogen receptor
GO	Gene ontology
FDR	False discovery rate
LNM	Lymph node metastases
FC	Fold change
OS	Overall survival
TCGA	The Cancer Genome Atlas
TACCO	Transcriptome Alterations in CanCer Omnibus
KEGG	Kyoto Encyclopedia of Genes and Genomes
PPI	Protein–protein interaction
DEGs	Differentially expressed genes
HR	Hazard ratio
OR	Odds ratio
