# Supplementary material for: Bioinformatics Analysis of microRNAs Associated with Metastatic Potential in Breast Cancer
Source: Biology (Basel). 2026 Apr 14;15(8):617. doi: 10.3390/biology15080617 (PMC13113051; doi:10.3390/biology15080617)
Supplement: Supplementary file 1 [file biology-15-00617-s001.zip › biology-4225509-supplementary.pdf]

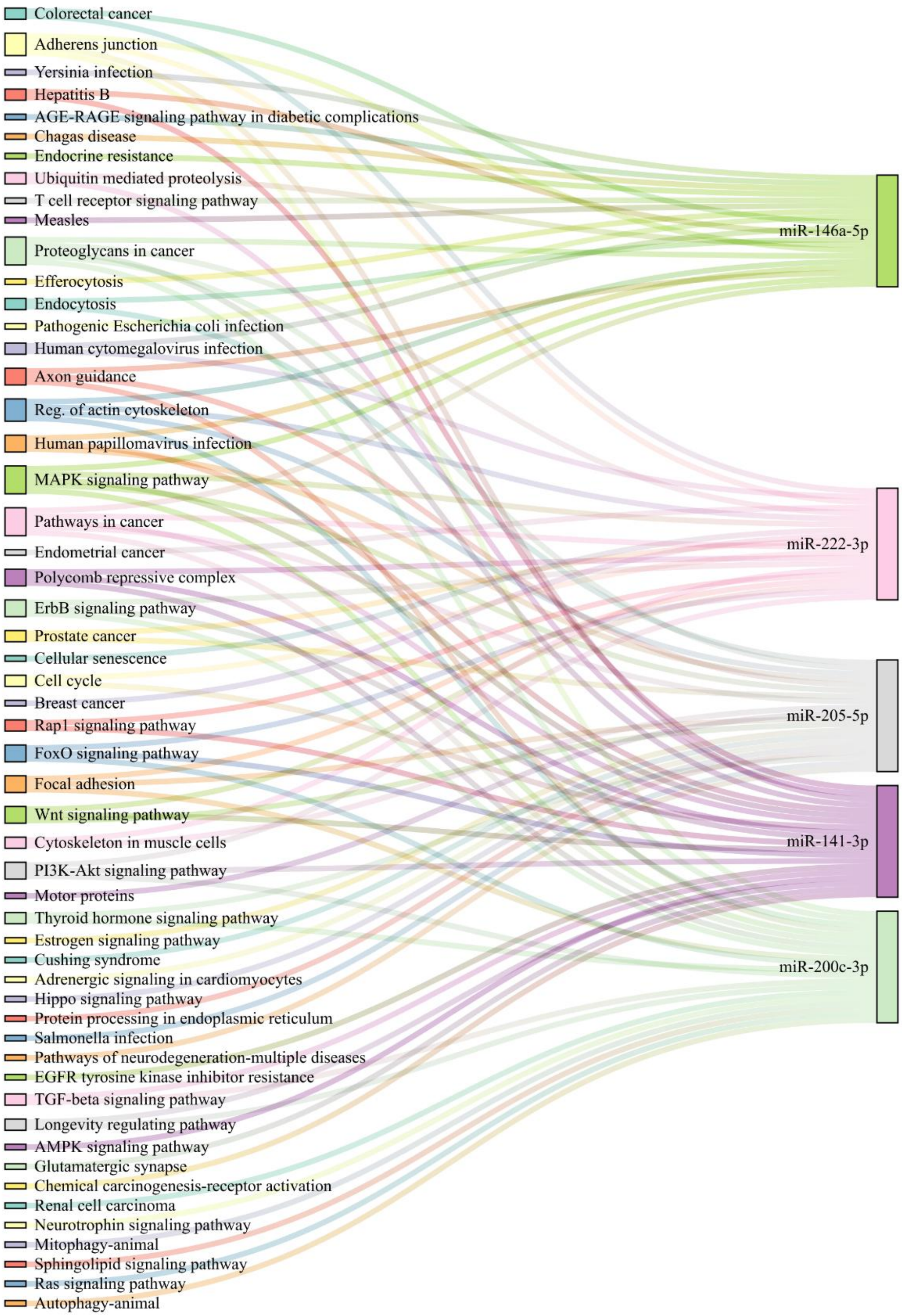

Suppl. Figure S1. Sankey plot depicting the results of the KEGG pathway enrichment analysis results (top 20 enriched terms) for putative target lists of miR-146a-5p, miR-222-3p, miR-205-5p, miR-141-3p and miR-200c-3p. Sizes of the bars assigned to KEGG pathway terms correspond to the number of connections with specific microRNA on the right side of the panel (shared enriched terms). Figure was constructed using https://www.chiplot.online/.

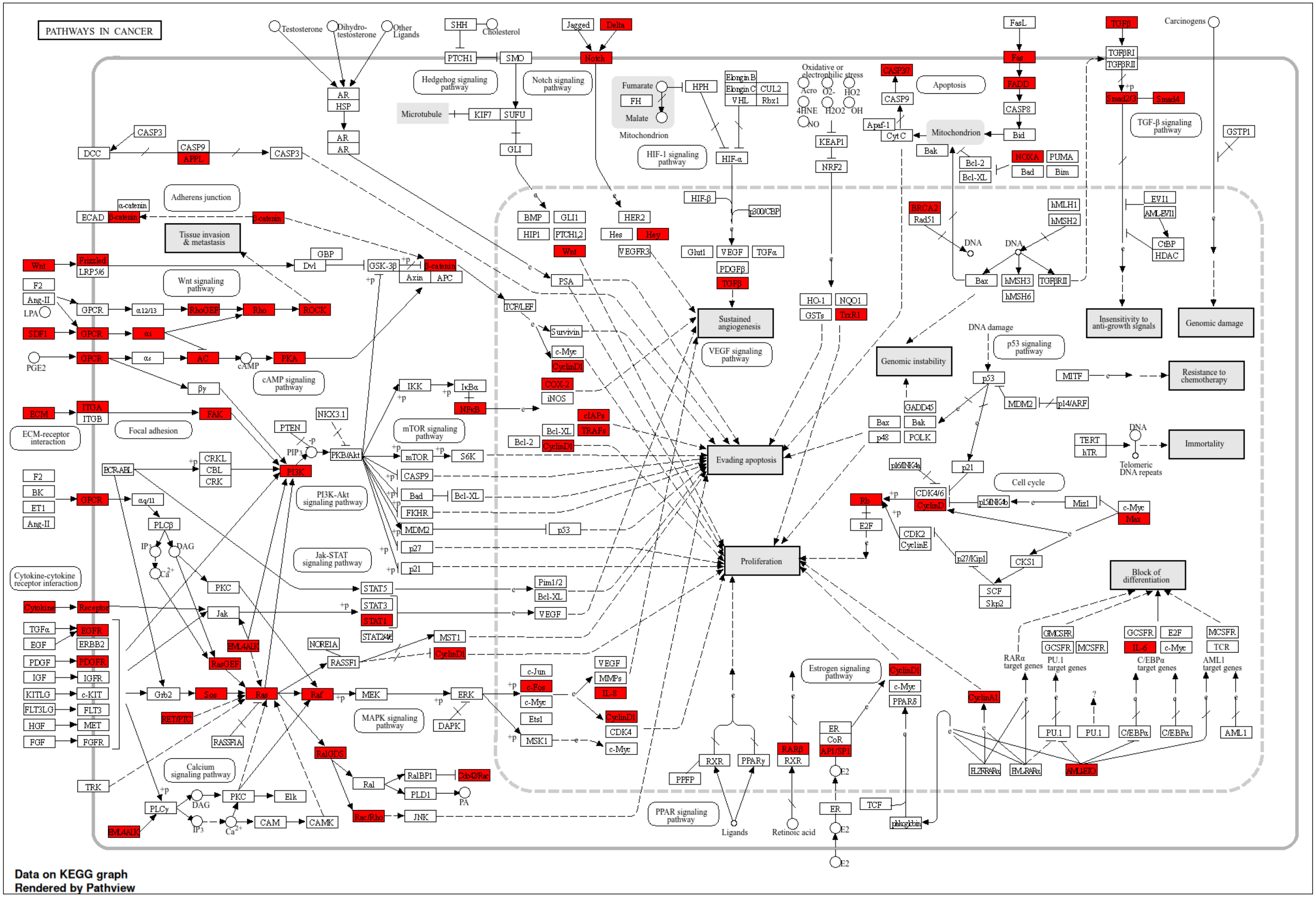

Suppl. Figure S2. KEGG pathway graph for “pathways in cancer” term with putative targets of miR-146a-5p highlighted in red.

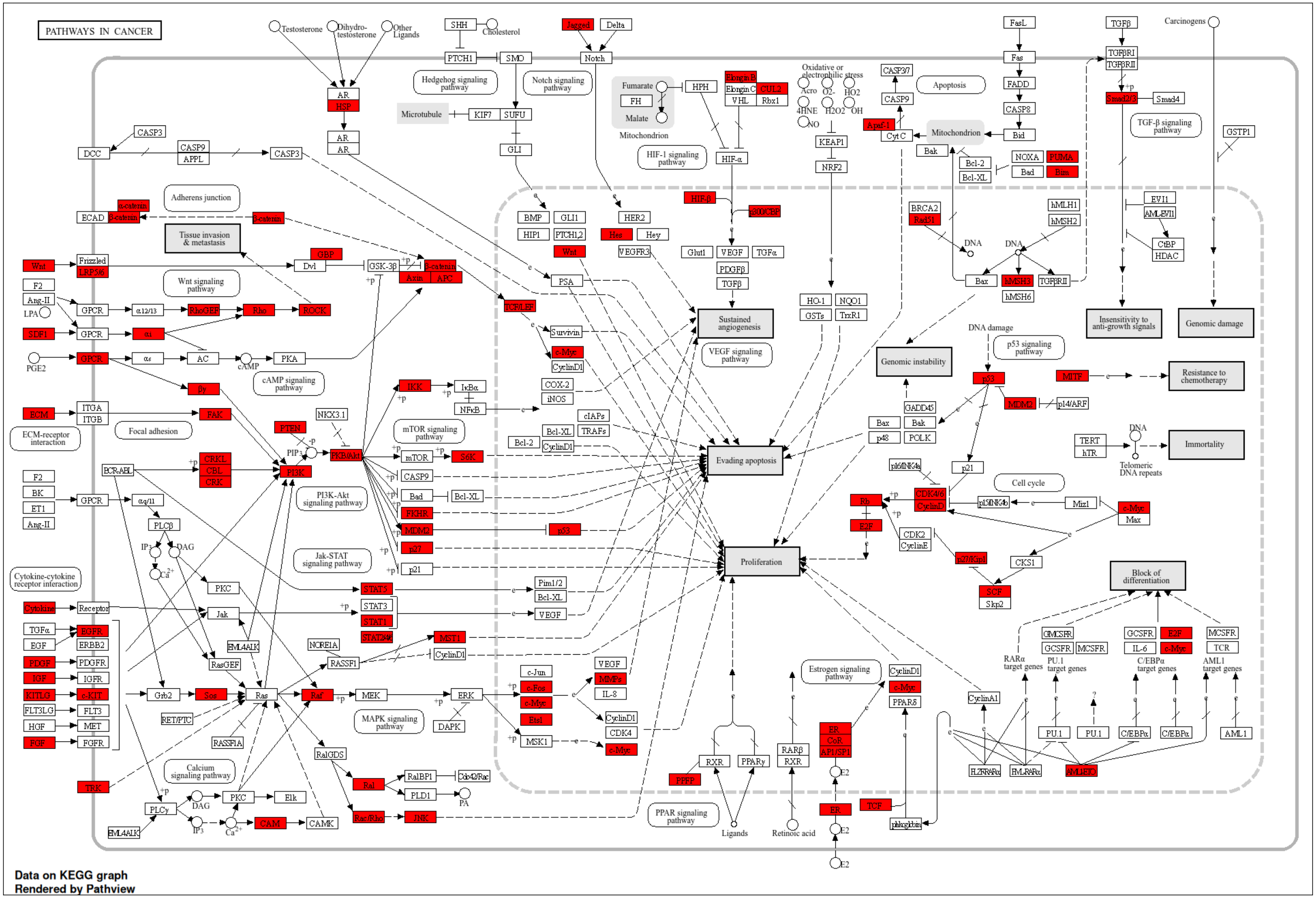

Suppl. Figure S3. KEGG pathway graph for “pathways in cancer” term with putative targets of miR-222-3p highlighted in red.

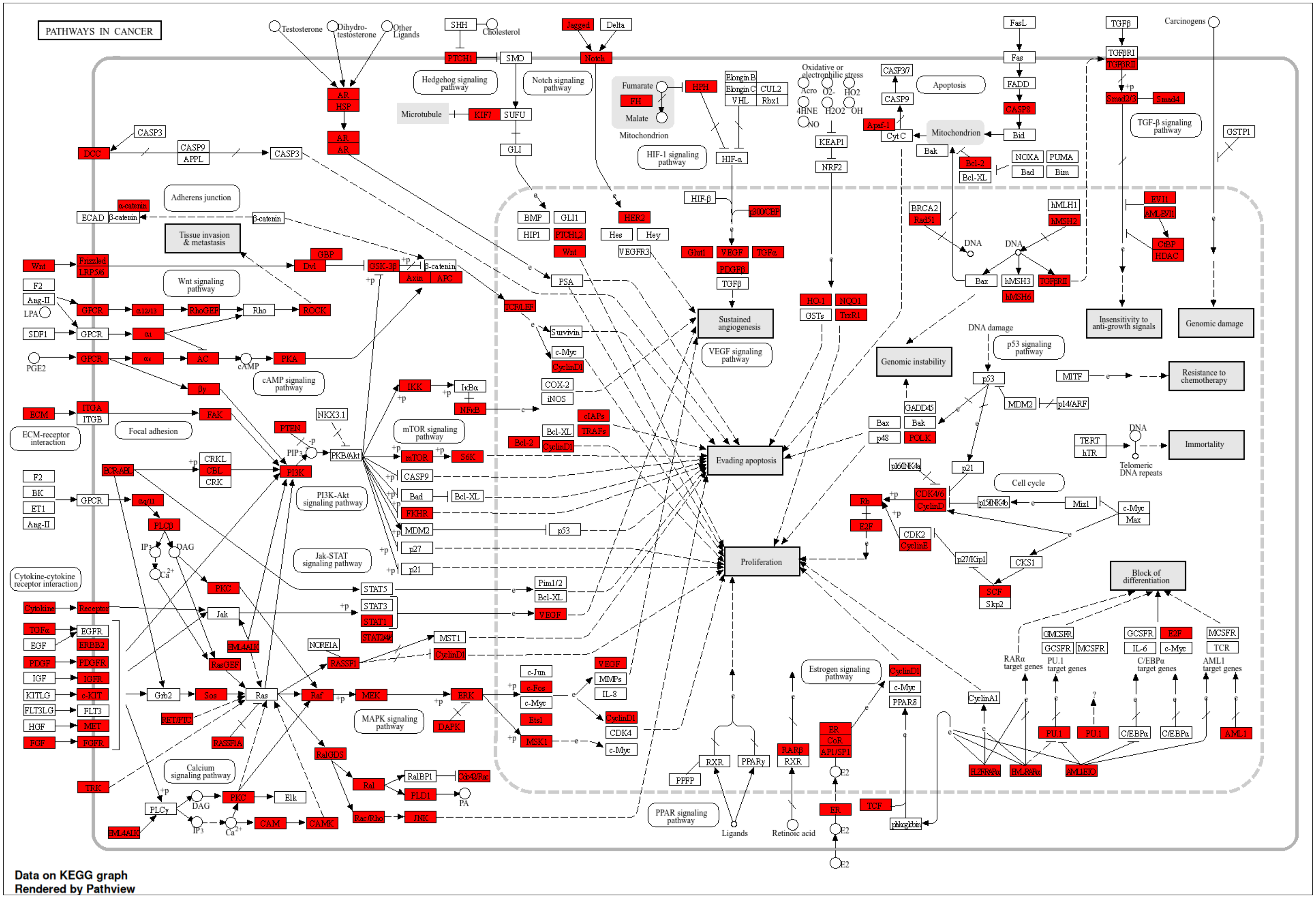

Suppl. Figure S4. KEGG pathway graph for “pathways in cancer” term with putative targets of miR-205-5p highlighted in red.

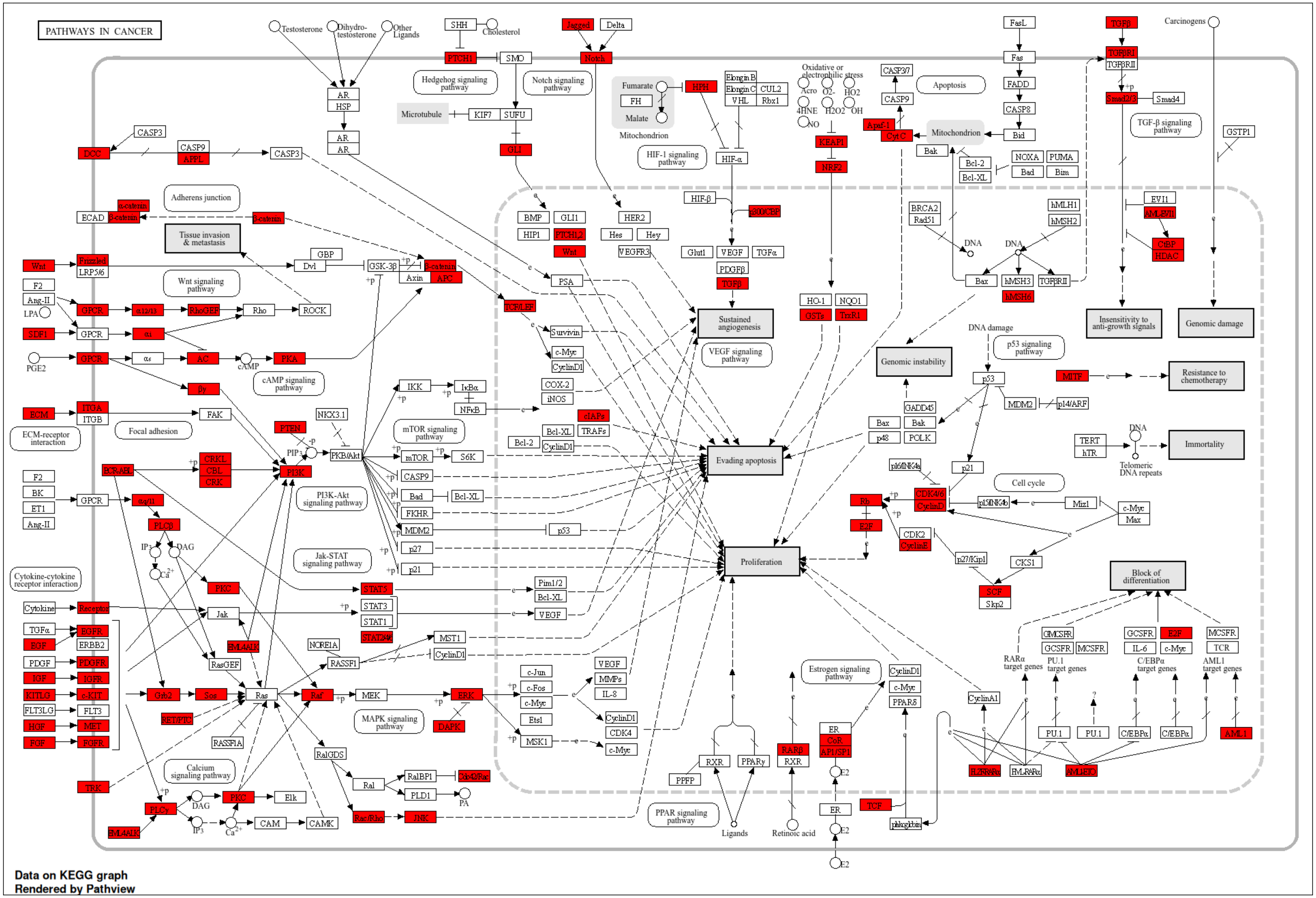

Suppl. Figure S5. KEGG pathway graph for “pathways in cancer” term with putative targets of miR-141-3p highlighted in red.

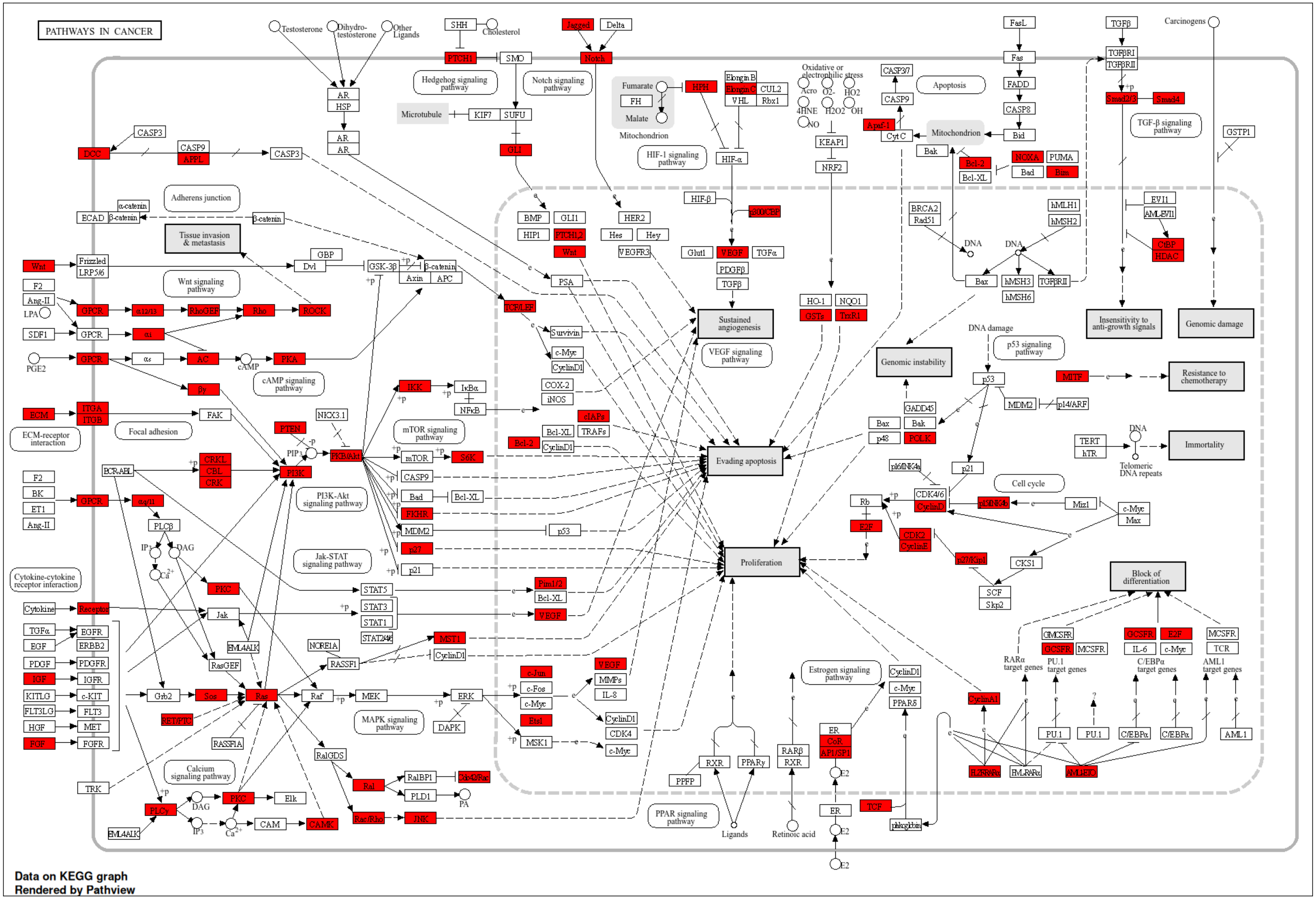

Suppl. Figure S6. KEGG pathway graph for “pathways in cancer” term with putative targets of miR-200c-3p highlighted in red.

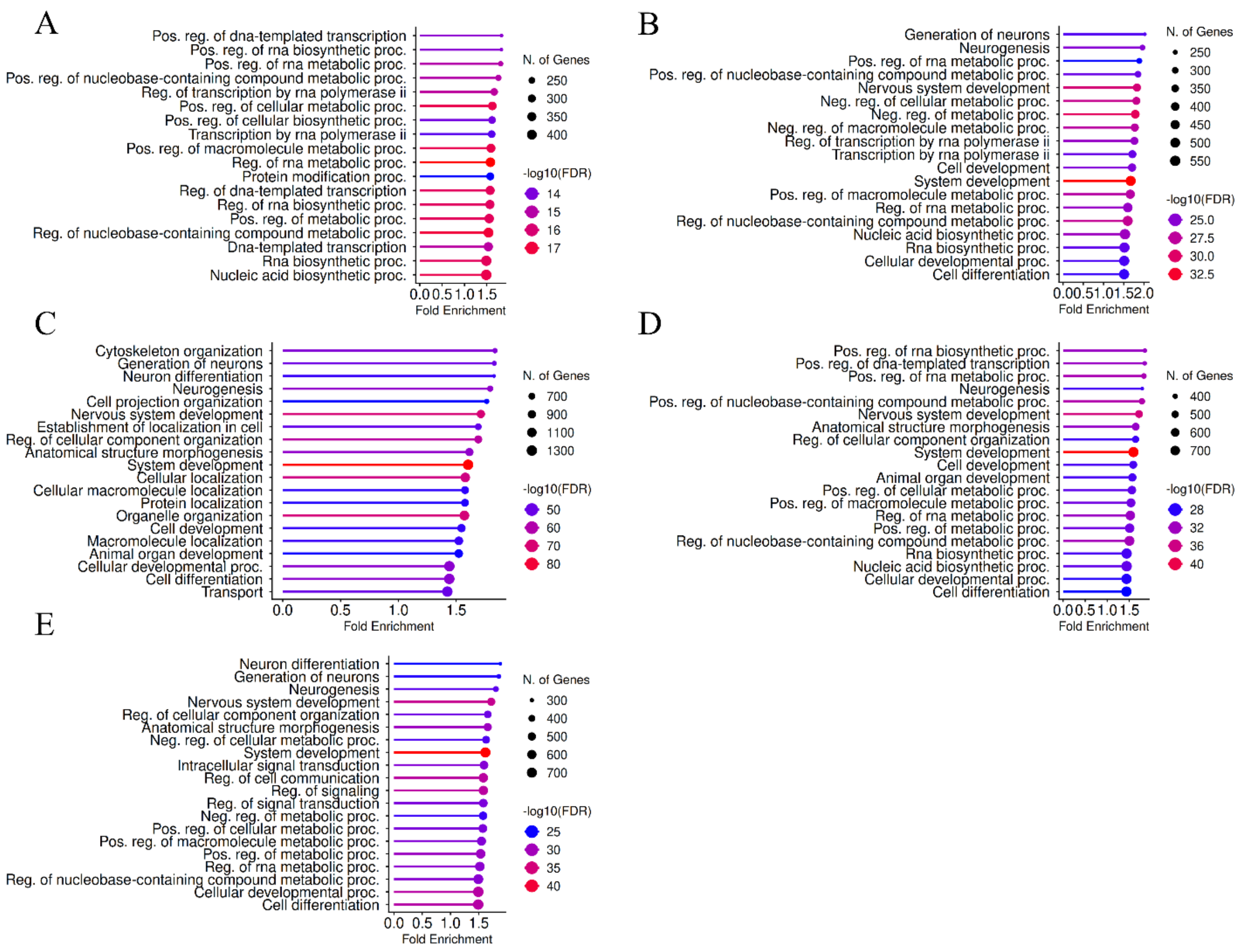

Supplementary Figure S7. Graphical representation of GO BP enrichment analysis results for putative target lists of (A) miR-146a-5p, (B) miR-222-3p, (C) miR-205-5p, (D) miR-141-3p and (E) miR-200c-3p. Terms were ranked according to fold enrichment, while the size of the circles corresponded to the number of genes and the color represented enrichment FDR values.

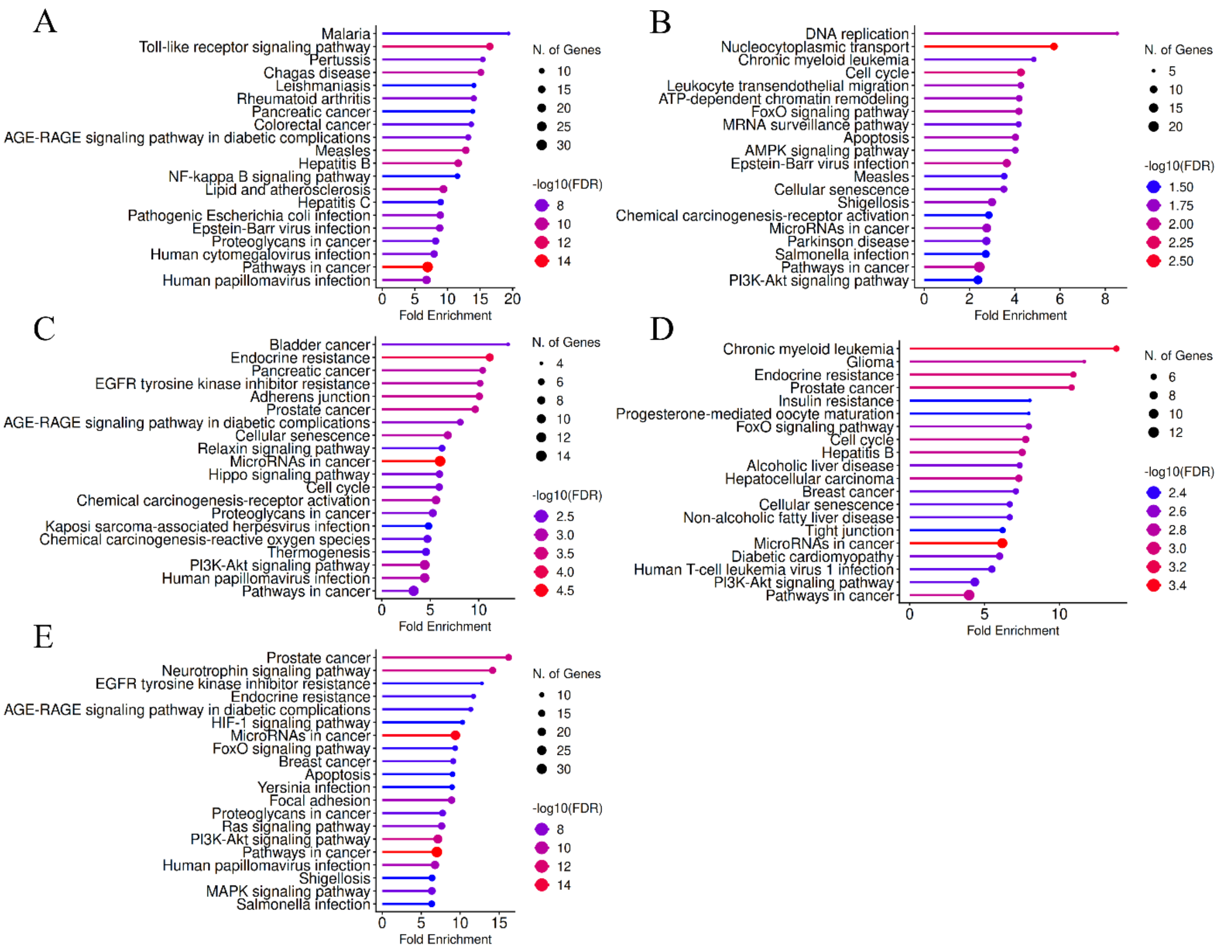

Supplementary Figure S8. Graphical representation of KEGG pathway enrichment analysis results for experimentally validated targets of (A) miR-146a-5p, (B) miR-222-3p, (C) miR-205-5p, (D) miR-141-3p and (E) miR-200c-3p. Terms were ranked according to fold enrichment, while the size of the circles corresponded to the number of genes and the color represented enrichment FDR values.

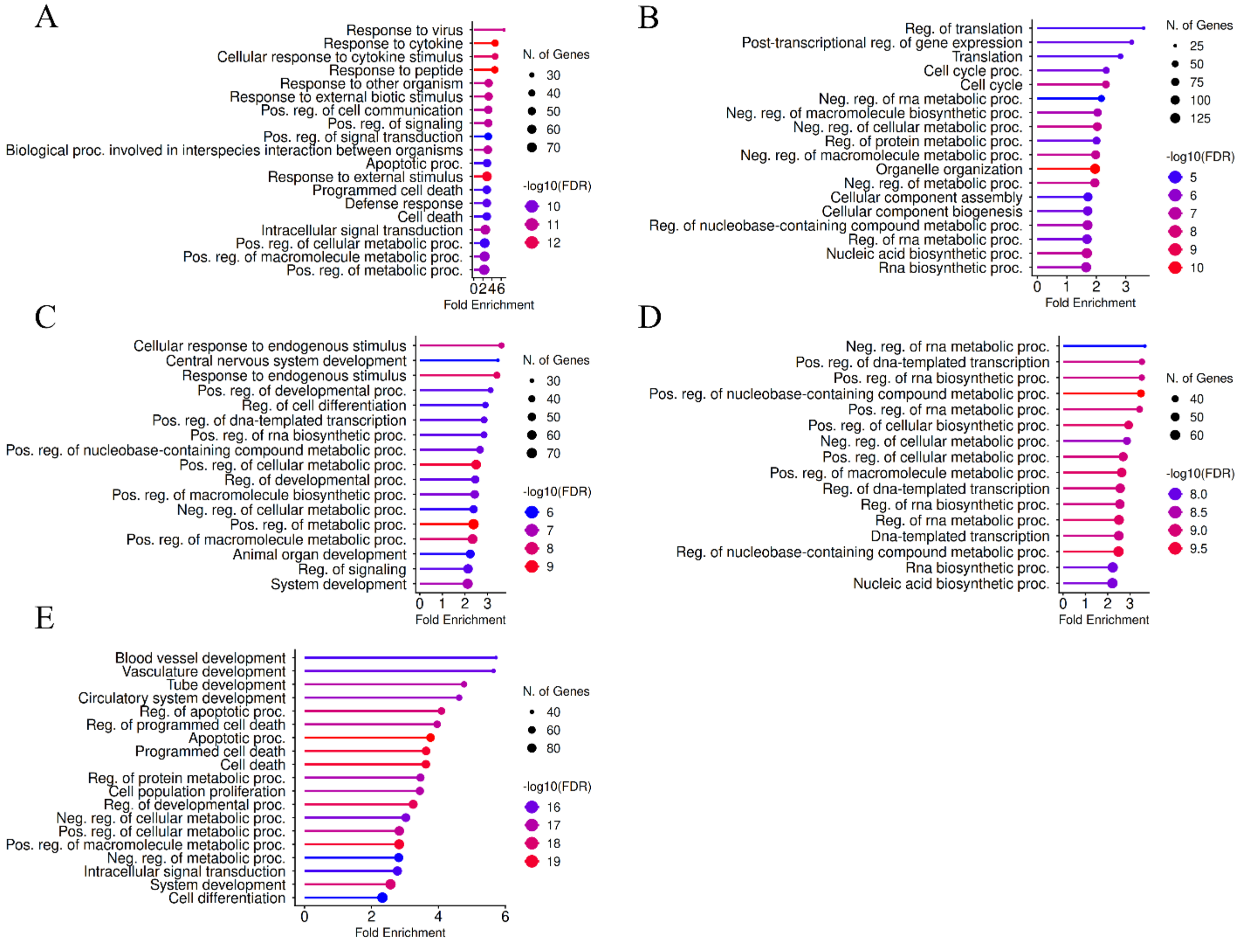

Supplementary Figure S9. Graphical representation of GO BP enrichment analysis results for experimentally validated targets of (A) miR-146a-5p, (B) miR-222-3p, (C) miR-205-5p, (D) miR-141-3p and (E) miR-200c-3p. Terms were ranked according to fold enrichment, while the size of the circles corresponded to the number of genes and the color represented enrichment FDR values.

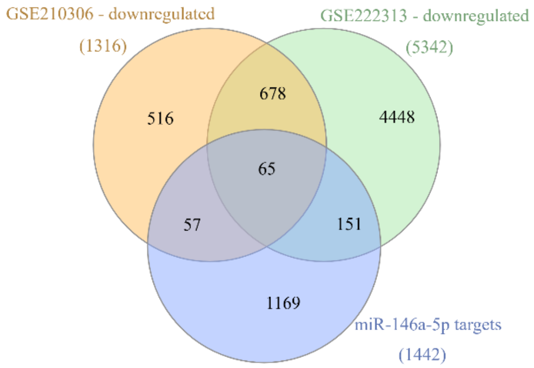

Supplementary Figure S10. Venn diagram depicting the intersection of upregulated DEGs from MDA-MB-231 vs. MCF7 comparison (GEO datasets GSE210306 and GSE222313) with putative targets of miR-146a-5p.

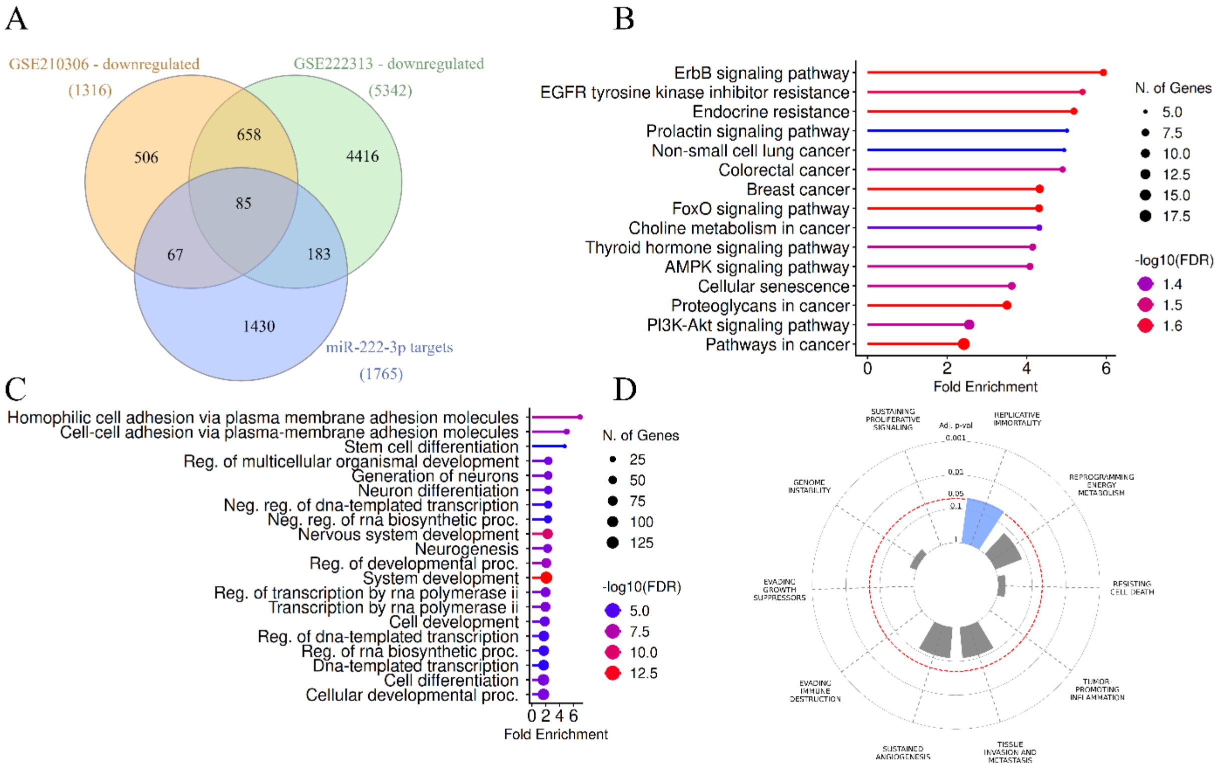

Supplementary Figure S11. Analysis of DEGs present within target list corresponding to miR-222-3p. A) Venn diagram depicting the cross section of downregulated DEGs from MDA-MB-231 vs. MCF7 comparison (GEO datasets GSE210306 and GSE222313) with putative targets of miR-222-3p; B) Graphical representation of KEGG pathway enrichment analysis; C) Graphical representation of GO BP enrichment analysis; D) Cancer hallmarks enrichment graph.

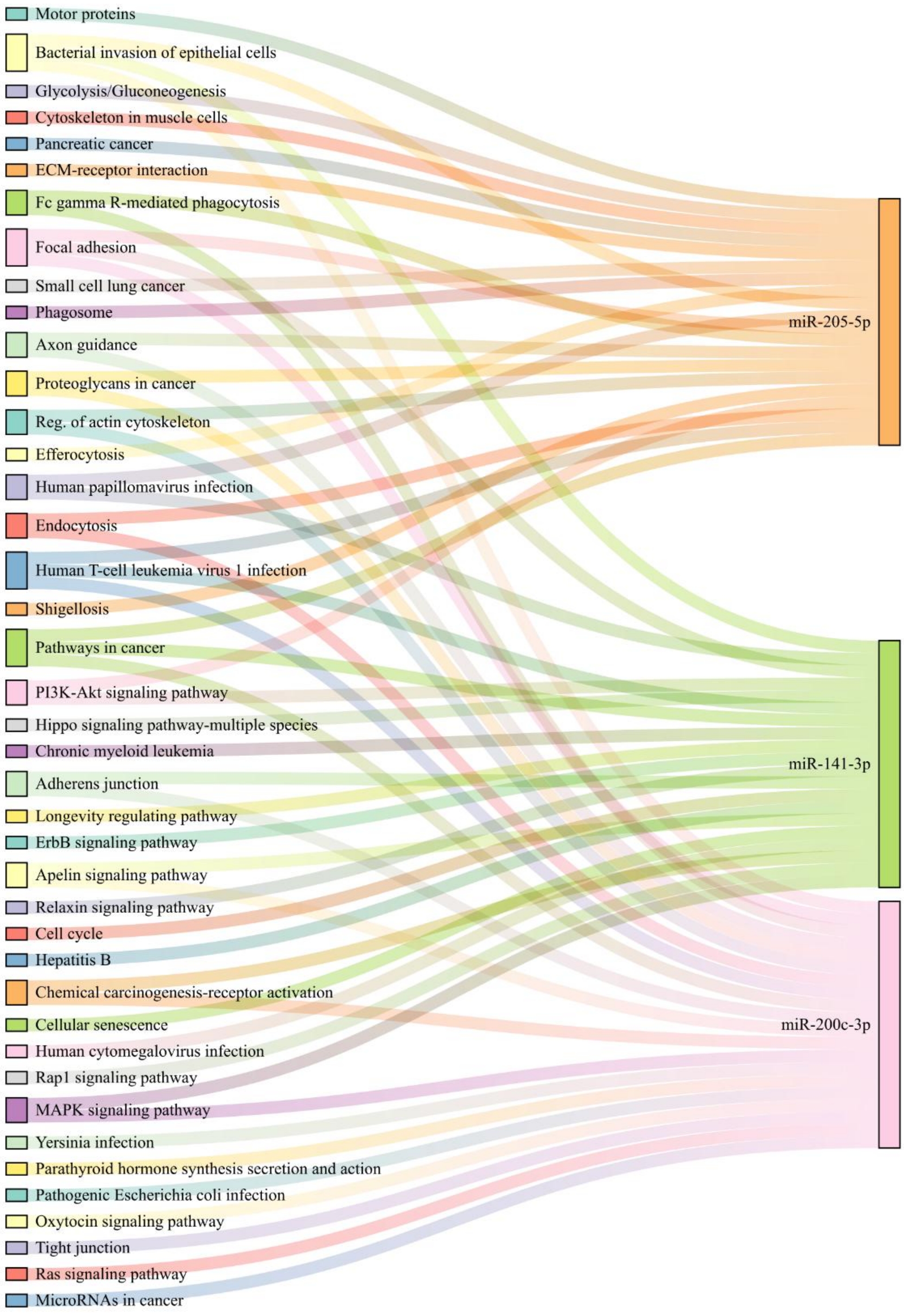

Suppl. Figure S12. Sankey plot depicting the results of the KEGG pathway enrichment analysis results (top 20 enriched terms) for DEGs among putative targets of miR-205-5p, miR-141-3p and miR-200c-3p. Sizes of the bars assigned to KEGG pathway terms correspond to the number of connections with specific microRNA on the right side of the panel (shared enriched terms). Figure was constructed using https://www.chiplot.online/.

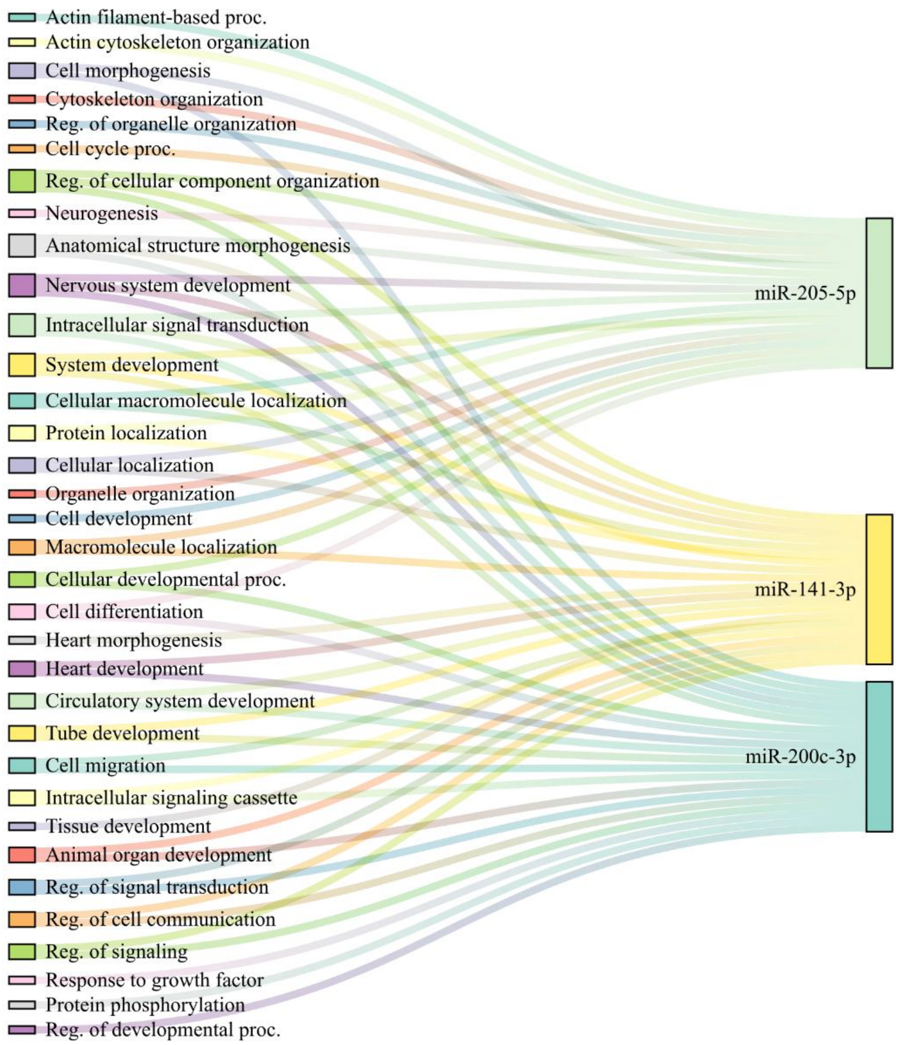

Suppl. Figure S13. Sankey plot depicting the results of the GO BP enrichment analysis results (top 20 enriched terms) for DEGs among putative targets of miR-205-5p, miR-141-3p and miR-200c-3p. Sizes of the bars assigned to GO BP terms correspond to the number of connections with specific microRNA on the right side of the panel (shared enriched terms). Figure was constructed using https://www.chiplot.online/.
